# Gene duplications facilitate C_4_-CAM compatibility in common purslane

**DOI:** 10.1093/plphys/kiad451

**Published:** 2023-08-17

**Authors:** Xiaoliang Wang, Xuxu Ma, Ge Yan, Lei Hua, Han Liu, Wei Huang, Zhikai Liang, Qing Chao, Julian M Hibberd, Yuannian Jiao, Mei Zhang

**Affiliations:** Institute of Botany, Chinese Academy of Sciences, Beijing 100093, China; State Key Laboratory of Plant Diversity and Specialty Crops, Beijing 100093, China; University of Chinese Academy of Sciences, Beijing 100049, China; China National Botanical Garden, Beijing 100093, China; Institute of Botany, Chinese Academy of Sciences, Beijing 100093, China; University of Chinese Academy of Sciences, Beijing 100049, China; China National Botanical Garden, Beijing 100093, China; Key Laboratory of Plant Molecular Physiology, Institute of Botany, Chinese Academy of Sciences, Beijing 100093, China; Institute of Botany, Chinese Academy of Sciences, Beijing 100093, China; University of Chinese Academy of Sciences, Beijing 100049, China; China National Botanical Garden, Beijing 100093, China; Key Laboratory of Plant Molecular Physiology, Institute of Botany, Chinese Academy of Sciences, Beijing 100093, China; Department of Plant Sciences, University of Cambridge, Cambridge CB2 3EA, UK; Institute of Botany, Chinese Academy of Sciences, Beijing 100093, China; China National Botanical Garden, Beijing 100093, China; Key Laboratory of Plant Molecular Physiology, Institute of Botany, Chinese Academy of Sciences, Beijing 100093, China; National Maize Improvement Center, China Agricultural University, Beijing 100193, China; Department of Plant and Microbial Biology, University of Minnesota, Saint Paul, MN 55108, USA; Institute of Botany, Chinese Academy of Sciences, Beijing 100093, China; China National Botanical Garden, Beijing 100093, China; Photosynthesis Research Center, Key Laboratory of Photobiology, Institute of Botany, Chinese Academy of Sciences, Beijing 100093, China; Department of Plant Sciences, University of Cambridge, Cambridge CB2 3EA, UK; Institute of Botany, Chinese Academy of Sciences, Beijing 100093, China; State Key Laboratory of Plant Diversity and Specialty Crops, Beijing 100093, China; University of Chinese Academy of Sciences, Beijing 100049, China; China National Botanical Garden, Beijing 100093, China; Institute of Botany, Chinese Academy of Sciences, Beijing 100093, China; University of Chinese Academy of Sciences, Beijing 100049, China; China National Botanical Garden, Beijing 100093, China; Key Laboratory of Plant Molecular Physiology, Institute of Botany, Chinese Academy of Sciences, Beijing 100093, China

## Abstract

Common purslane (*Portulaca oleracea*) integrates both C_4_ and crassulacean acid metabolism (CAM) photosynthesis pathways and is a promising model plant to explore C_4_-CAM plasticity. Here, we report a high-quality chromosome-level genome of nicotinamide adenine dinucleotide (NAD)-malic enzyme (ME) subtype common purslane that provides evidence for 2 rounds of whole-genome duplication (WGD) with an ancient WGD (P-β) in the common ancestor to Portulacaceae and Cactaceae around 66.30 million years ago (Mya) and another (Po-α) specific to common purslane lineage around 7.74 Mya. A larger number of gene copies encoding key enzymes/transporters involved in C_4_ and CAM pathways were detected in common purslane than in related species. Phylogeny, conserved functional site, and collinearity analyses revealed that the Po-α WGD produced the phosphoenolpyruvate carboxylase-encoded gene copies used for photosynthesis in common purslane, while the P-β WGD event produced 2 ancestral genes of functionally differentiated (C_4_- and CAM-specific) beta carbonic anhydrases involved in the C_4_ + CAM pathways. Additionally, cis-element enrichment analysis in the promoters showed that CAM-specific genes have recruited both evening and midnight circadian elements as well as the Abscisic acid (ABA)-independent regulatory module mediated by ethylene-response factor cis-elements. Overall, this study provides insights into the origin and evolutionary process of C_4_ and CAM pathways in common purslane, as well as potential targets for engineering crops by integrating C_4_ or CAM metabolism.

## Teaser

Common purslane has undergone 2 rounds of whole-genome duplication (WGD), and the ancient P-β WGD produced the ancestral genes of functional C_4_- and crassulacean acid metabolism (CAM)-specific gene copies involved in C_4_ + CAM pathways.

## Introduction

Common purslane (*Portulaca oleracea*), a member of the Portulacaceae family in the Caryophyllales, is an annual herb that is widely dispersed over the world. Based on numerous morphological and chromosome number variations, it has been described as either a polymorphic species or a complex of subspecies (Ocampo and Columbus 2012; Rice et al. 2015; Walter et al. 2015; Ferrari et al. 2020b). Common purslane is a medicinal and edible plant, is rich in nutrients, and accumulates carotene, vitamin C, vitamin E, and ω-3 unsaturated fatty acids (Omara-Alwala et al. 1991; Simopoulos et al. 1992; Uddin et al. 2014). Notably, common purslane can tolerate extremely high temperatures combined with drought, high humidity, high salt, and low nutrient levels (Zimmerman 1976; Yang et al. 2012; D'Andrea et al. 2014). Together, these characteristics make *P. oleracea* an attractive model for both basic and applied research.

Common purslane has developed multiple resistance strategies to abiotic stresses (Lara et al. 2004; Jin et al. 2016; Habibi 2020). One such example is its photosynthetic pathway that is responsive to drought—although it is a canonical C_4_ plant, common purslane performs facultative crassulacean acid metabolism (CAM) photosynthesis under drought conditions (Koch and Kennedy 1980; Lara et al. 2004; D'Andrea et al. 2014). Such an integrated C_4_-CAM system has been described in another purslane, Paraguayan purslane (*P. amilis*) (Gilman et al. 2022), but to our knowledge, this integration is rare in land plants. C_4_ and CAM both facilitate high-efficiency photosynthesis by concentrating CO_2_ near Rubisco (Hatch 1987; Edwards and Ogburn 2012) to generate so-called CO_2_-concentrating mechanisms (CCMs). These 2 metabolisms share a series of biochemical reactions and enzymes (Ferrari and Freschi 2019). CO_2_ is incorporated into oxaloacetate by the concerted action of beta carbonic anhydrase (β-CA) and then phosphoenolpyruvate carboxylase (PEPC) during initial carboxylation, followed by its release from malate or aspartate via nicotinamide adenine dinucleotide phosphate (NAD(P))-malic enzyme (ME)–mediated decarboxylation before entering the Calvin cycle. The major difference between the C_4_ and CAM pathway is the spatial separation of carboxylation and decarboxylation associated with the C_4_ pathway—carboxylation takes place in mesophyll cells (MCs) and decarboxylation in bundle sheath cells—while in CAM, these 2 processes are separated temporally in MCs.

Recently, Moreno-Villena et al. demonstrated that CAM and C_4_ carbon fixation occur in the same cells and that CAM-generated metabolites are likely directly incorporated into the C_4_ cycle in common purslane (Hibberd 2022; Moreno-Villena et al. 2022). Based on phylogenetic analysis of PEPC, a key enzyme for C_4_ and CAM metabolism in the genus *Portulaca*, *PEPC1E1* (*ppc-1E1*) genes were proposed to be recruited into both C_4_ and CAM photosynthesis in the Caryophyllales (Christin et al. 2014; Goolsby et al. 2018; Moore et al. 2018). Subsequently, several studies found that PEPC is encoded by many gene copies in *Portulaca*, and although it has not been possible to test the importance of all PEPC gene copies for photosynthesis, it was proposed that one copy (*PPC-1E1a′*) is specific for C_4_ and another (*PPC-1E1c*) for CAM in both common purslane and Paraguayan purslane (Ferrari et al. 2019; Gilman et al. 2022; Moreno-Villena et al. 2022). The *PPC-1E1a′* copy is highly expressed during the daytime in well-watered plants, while the *PPC-1E1c* copy displays the highest expression during the day-night transition in plants under drought stress (Christin et al. 2014; Ferrari et al. 2019; Gilman et al. 2022; Moreno-Villena et al. 2022). However, it is still unknown whether and how duplication events are associated with these C_4_- and CAM-specific PEPC gene copies as well as others encoding essential enzymes or transporters used in C_4_ and CAM.

The origin of C_4_ and CAM in *Portulaca* has not been comprehensively studied. Facultative CAM is assumed to be ancestral in *Portulaca* because it has been observed in every major subclade as well as *Portulaca*'s closest relatives (Anacampserotaceae and Cactaceae) (Sage 2002; Christin et al. 2014; Gilman et al. 2022). This hypothesis was supported from an analysis of the evolutionary history of PEPC genes, with CAM-specific copies of *Portulaca* being similar to CAM forms of other species in Cactaceae (Christin et al. 2014). However, this approach has not been possible to take for other genes important for C_4_ and CAM such as β-CA. A high-quality genome assembly of *Portulaca* species along with published genomes of *Portulaca*'s closest relatives (e.g. dragon fruit [*Hylocereus undatus*] in Cactaceae) would allow us to comprehensively explore mechanisms, allowing integration of the C_4_ and CAM pathways. A de novo genome assembly for Paraguayan purslane from Illumina short reads was reported recently. It indeed provided a scaffold level genome and revealed evidence of coexpression networks supporting C_4_-CAM compatibility (Gilman et al. 2022). However, the origin process of C_4_ and CAM and the importance of whole-genome duplication (WGD) events in the evolutionary history of the Portulacaceae remain to be explored.

Here, we report a high-quality chromosome-level genome assembly for common purslane, which revealed 2 rounds of WGD: the ancient WGD (hereafter abbreviated as the P-β) in common purslane is shared with dragon fruit, while an independent WGD (hereafter abbreviated as the Po-α) occurred in common purslane. These WGD events and tandem duplication (TD) events produced multiple copies of genes encoding key enzymes/transporters of C_4_ photosynthesis and CAM. We identified 4 C_4_-specific *PEPC* genes from Po-α WGD/TD and 2 CAM-specific PEPC genes from Po-α WGD based on conserved functional sites and diurnal expression patterns. Importantly, we found that the P-β WGD event produced 2 ancestral genes of functionally differentiated (C_4_- and CAM-specific) β-CA genes involved in C_4_ + CAM pathways by phylogenetic trees and evolutionary history. Motif enrichment analysis in promoters of CAM-specific genes showed enriched motif clusters but little overlap with night or drought-specific promoters, suggesting facultative CAM results from the recruitment of complex and independent regulatory networks. Thus, this study not only provides further evidence of C_4_-CAM compatibility in one leaf but also sheds light on the origin and evolution of C_4_ and CAM in *Portulaca* and the integration of CAM and/or C_4_ metabolism, which is important for biotechnological applications of these pathways.

## Results

### Genome assembly and annotation of common purslane

**Figure 1.**
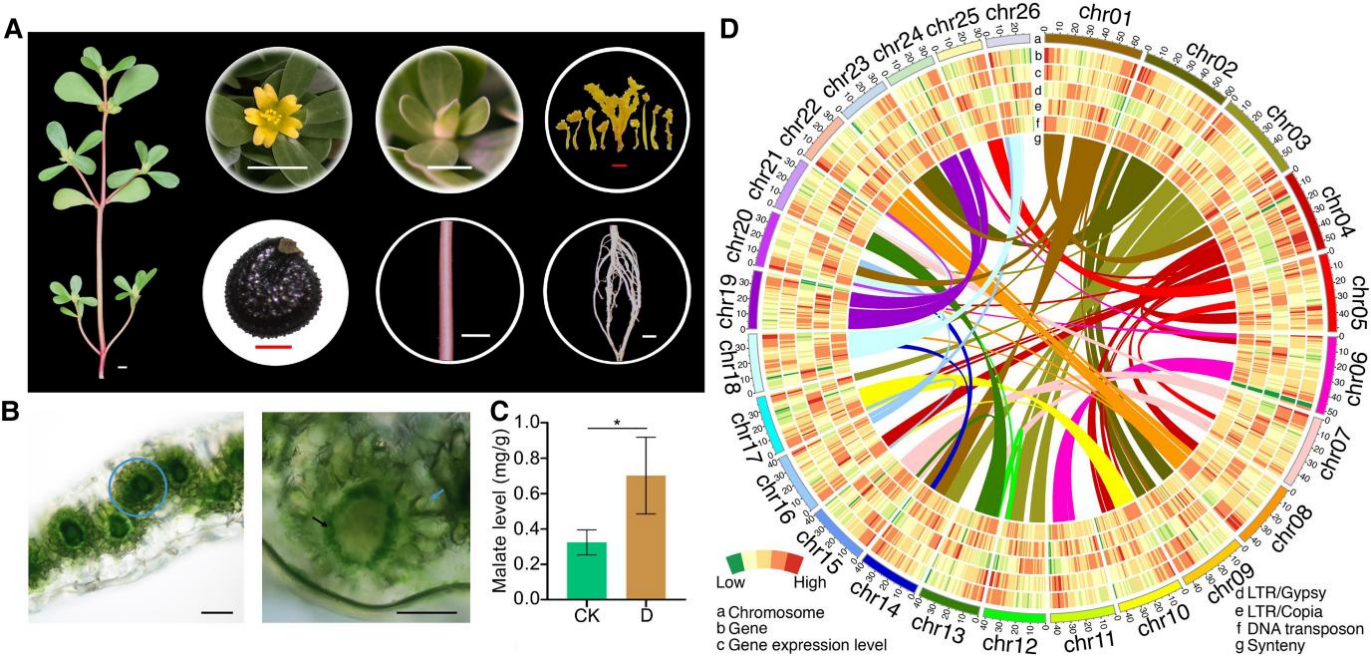
Overview of the common purslane genome assembly and features. **A)** Morphology of the common purslane seedling, flowers, separated pistil and stamen, mature seeds, stem, and root. Images were digitally extracted for comparison. White scale bars in flowers, leaf, stem, and root: 0.5 cm; red scale bars in separated pistil and stamen, and mature seeds: 0.2 mm. **B)** Handmade leaf slice showing the C_4_ Kranz anatomy of common purslane leaves. Blue circle shows typical Kranz leaf anatomy. Black and blue arrows show bundle sheath and MCs, respectively. Scale bars, 0.1 mm. **C)** Malate accumulation in common purslane leaves under normal or drought conditions at night. Data are means ± Se of 3 biological replicates. *P < 0.05 by *t*-test. **D)** Overview of the common purslane genome assembly. Track a, 26 assembled chromosomes; Tracks b to f represent the other genomic features as indicated at the lower left of the circle plot. The colors indicate the density of genomic features in 1-Mb sliding window along chromosomes. Track g shows syntenic blocks. Band width is proportional to the size of the syntenic block. CK, control; D, drought.

We selected a wild purslane plant growing in the field (Beijing, China) for genome sequencing and assembly. Using plant taxonomy indicators, we confirmed that this wild purslane was the common purslane (Fig. 1A). It had typical Kranz anatomy (Fig. 1B), corresponding to C_4_ photosynthesis. Increased level of malate accumulation was also observed at night under drought conditions (Fig. 1C), which indicates nocturnal CO_2_ fixation occur. Overall, these characters demonstrated that common purslane we used also performs both C_4_ and CAM photosynthesis pathways as other *Portulaca* species (Voznesenskaya et al. 2010, 2017; Gilman et al. 2022).

To examine the karyotype of this common purslane, we utilized 2-color fluorescence in situ hybridization (FISH) with *5S* rDNA and *45S* rDNA as probes and detected 52 chromosomes (Supplemental Fig. S1). This result was consistent with the analysis of wild common purslane widely grown in China but different from the common purslane found in other countries used in previous studies (Ocampo and Columbus 2012; Walter et al. 2015). We estimated genome size to 1,122 Mb based on *k*-mer analysis of Illumina short reads (Supplemental Fig. S2), which agreed with the estimation of 1,137 Mb obtained by flow cytometry (Supplemental Fig. S2). The sequenced common purslane individual exhibited low heterozygosity, with overall genome heterozygosity of 0.059% (Supplemental Fig. S2).

We built a de novo assembly of the common purslane genome using 4 different sequencing technologies (Supplemental Fig. S3). The first draft assembly consisted of 201 contigs, spanning 1,119 Mb, derived from 80× coverage with Nanopore long reads (read N50 = 29 kb; contig N50 = 18.23 Mb; Table 1). We then integrated optical mapping data, yielding an assembly of 1,134 Mb with 101 scaffolds (N50 of 35.10 Mb), covering 99.74% of the estimated genome size based on flow cytometry and *k*-mer analyses (Supplemental Table S1). Finally, we ordered and oriented the scaffolds into 26 pseudochromosomes using High-resolution chromosome conformation capture (Hi-C) data (Fig. 1D and Supplemental Fig. S4). Notably, the longest contig spanned the entire length of chromosome 16. We assessed the completeness and quality of the genome assembly with several strategies. First, we generated transcriptome deep-sequencing (RNA-seq) data from 7 different tissues and resequenced the common purslane genome via Illumina short reads. We then mapped the RNA-seq, DNA-seq data, and Nanopore long reads onto the final assembly with mapping rates of 96.47%, 99.39%, and 99.44%, respectively (Supplemental Table S2). Second, the complete benchmarking universal single copy orthologs (BUSCOs) and consensus quality value (QV) of genome assembly were 96.8% (Supplemental Table S2) and 32.4, respectively. Third, we estimated the long terminal repeat (LTR) assembly index (LAI) of the common purslane genome to be 17.96, indicating that it shows the qualities of a “reference” genome (10 < LAI < 20) (Supplemental Fig. S5). Finally, we calculated the genome coverage using Nanopore long reads and Hi-C joins, yielding results of 98.1% and 99.7%, respectively. Overall, we obtained a high-quality genome in terms of genome completeness, continuity, and accuracy.

**Table 1.** Summary statistics of the common purslane genome assembly and annotation

Genomic features	Values
Estimated genome size	1,137 Mb
Assembled genome size	1,134 Mb
No. of contigs	201
N50 length of contigs	18.23 Mb
No. of scaffolds	101
N50 length of scaffolds	35.10 Mb
No. of pseudochromosomes	26
Length of sequence assigned to chromosomes	1,131 Mb
Percentage of sequence assigned to chromosomes	99.70%
Percentage of repeat sequences	67.64%
No. of annotated high-confidence genes	45,250
Complete BUSCOs (embryophyta_odb10)	98.00%
Percentage of gene with functional annotation	91.90%

Using a combination of plant homology searches, transcriptome-based predictions, and ab initio gene predictions, we identified 45,250 high-confidence, protein-coding genes, corresponding to a BUSCO score of 98.00% for complete genes (single-copy and duplicated; Supplemental Table S3). We obtained potential functional annotation information for 41,585 (91.90%) genes using the EuKaryotic Orthologous Groups, Kyoto Encyclopedia of Genes and Genomes, Nonredundant Protein Sequence Database, SwissProt, and Gene Ontology (GO) databases (Supplemental Table S4). Transposons made up 63.70% of the common purslane genome sequence, with LTR retrotransposons being the largest family, accounting for about 76.61% of all transposable elements (TEs) and representing 48.80% of the genome assembly (Supplemental Table S5). Among LTR retrotransposons, Ty3/*Gypsy* elements represented 23.71% of the genome and were much more numerous than Ty1/*Copia* elements, which covered only 3.94% of the genome (Supplemental Table S5). We observed a distinct unimodal distribution for the insertion times of intact LTR/*Gypsy* in the common purslane genome, with a peak of amplification around 0.5 million years ago (Mya), while the LTR/*Copia* showed a burst about 0.8 Mya (Supplemental Fig. S6). Like other plant genomes, the common purslane genome also exhibited high Ty3*/Gypsy* density in regions with low protein-coding gene density (Fig. 1D).

### Whole-genome duplications

**Figure 2.**
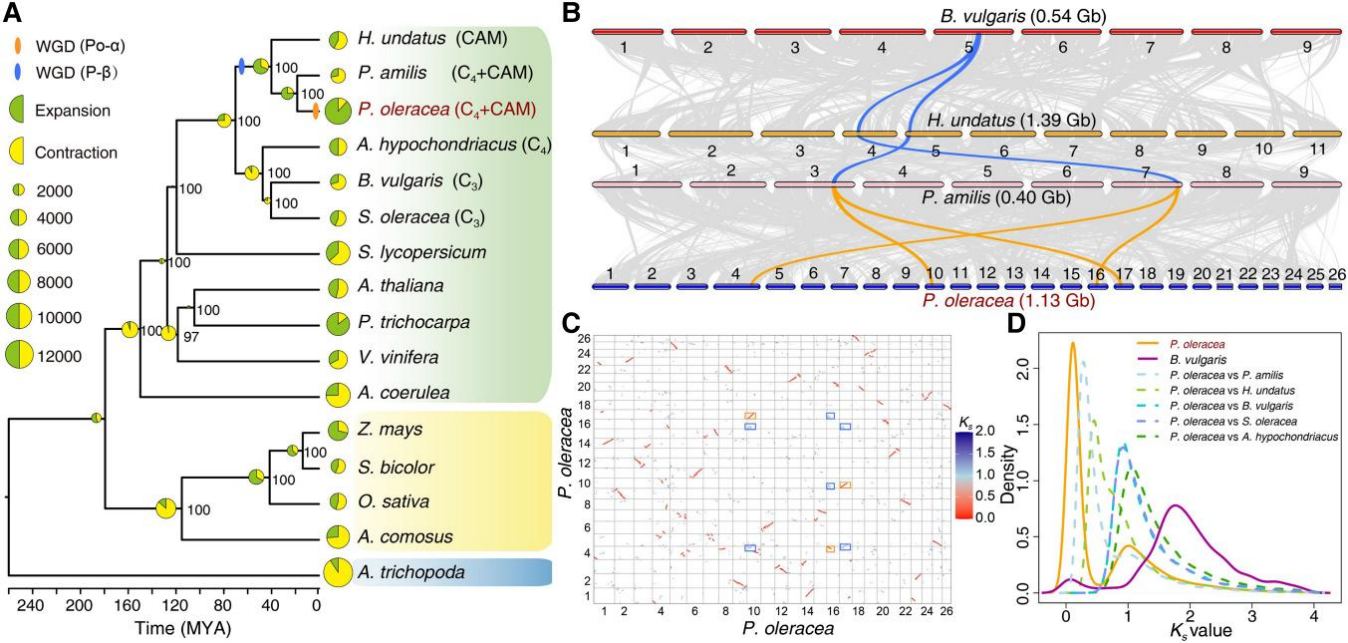
Evolutionary analysis of the common purslane genome. **A)** Phylogenetic species tree constructed based on single-copy putative orthologs. The lineage divergence times (Mya) and gene family expansion and contraction are shown. The divergence times were estimated by r8s (v1.81). The pie charts at each branch of the tree represent the proportion of gene families undergoing gain or loss events. The size of the pie charts is proportional to the number of gene families expanded (green) or contracted (yellow). The numbers at each branch represent the percentage support. **B)** Macrosynteny comparisons among *B. vulgaris* (0.54 Gb), *H. undatus* (1.39 Gb), *P. amilis* (0.40 Gb), and *P. oleracea* (1.13 Gb) revealing a 1:2:2:4 ratio, the region highlighted in orange provides 1 example. **C)** Syntenic dot plot of *P. oleracea* against itself. Syntenic gene pairs were colored as a function of the synonymous substitution rate (*K*_s_) values (*K*_s_ > 1, blue; *K*_s_ < 1, red). The recent and relatively ancient WGDs are highlighted by the orange and blue boxes, respectively. **D)***K*_s_ distribution in the identified syntenic putatively paralogous blocks from *P. oleracea* and *B. vulgaris* (solid lines), and *P. oleracea* putative orthologs with 5 other species (dashed lines).

To investigate the evolution of the common purslane genome, we included another 15 genomes from major angiosperm clades, comprising 10 eudicots, 4 monocots, and *Amborella* as an outgroup, for comparative genomic analyses. We utilized a set of 47 single-copy gene families from these 16 species to construct a phylogenetic tree (Fig. 2A). The resulting topology was consistent with that of the Angiosperm Phylogeny Group IV (Byng et al. 2016). We observed that the Portulacaceae (*P. oleracea* and *P. amilis*) cluster with Cactaceae (dragon fruit [*H. undatus*]) among the Caryophyllales. Further phylogenetic dating analysis indicated that *P. oleracea* (Portulacaceae) diverged from *H. undatus* (Cactaceae) around 40 Mya, while the clade containing *P. oleracea* and *H. undatus* diverged from the Amaranthaceae (spinach [*Spinacia oleracea*], sugar beet [*Beta vulgaris*], and amaranth [*Amaranthus hypochondriacus*]) around 70 Mya (Fig. 2A).

The high percentage of complete duplicated genes in the *P. oleracea* genome, as indicated by BUSCO analysis, suggested widespread duplication events. We therefore explored the evolutionary history of WGD events in the *P. oleracea* lineage by combining evidence from synonymous substitution rate (*K*_s_) and synteny analyses (Fig. 2, B to D). The most recent WGD event in *B. vulgaris* is the ancestral gamma hexaploidization (γ-WGD) shared by core eudicots (Dohm et al. 2013; Xu et al. 2017); we thus used the *B. vulgaris* genome as a reference to identify duplication events in the Caryophyllales. Syntenic analyses detected a clear 1:3 colinearity relationship when we compared the *P. oleracea* genome with itself (Fig. 2C), suggesting that 2 WGDs occurred in the evolutionary history of *P. oleracea.* Dotplots of syntenic gene pairs identified a 4:1 syntenic ratio between the *P. oleracea* and *B. vulgaris* genomes, a 4:2 syntenic ratio between *P. oleracea* and *H. undatus*, and a 4:2 syntenic ratio between *P. oleracea* and *P. amilis* (Supplemental Fig. S7). Macrosynteny patterns between genomic regions from *B. vulgaris*, *H. undatus*, *P. amilis*, and *P. oleracea* clearly showed a 1:2:2:4 ratio, supporting 2 rounds of WGD in *P. oleracea* after its divergence from Amaranthaceae and 1 WGD in the *H. undatus* lineage (Fig. 2B). To validate this WGD history, we plotted the *K*_s_ distribution of anchor gene pairs from intra- and intergenomic syntenic blocks. We observed 2 distinct *K*_s_ peaks in the *P. oleracea* genome (Fig. 2D), consistent with a genome history with 2 WGD events. Given the *K*_s_ peak of putatively orthologous gene pairs between *P. oleracea* and *P. amilis* (*K*_s_ = 0.28) and *H. undatus* (*K*_s_ = 0.45), we hypothesized that the P-β WGD in *P. oleracea* was shared with *H. undatus*, while the Po-α WGD occurred in *P. oleracea*. Together with evidence from synteny and *K*_s_ analyses, our results strongly suggested the P-β WGD be in the common ancestor to Portulacaceae and Cactaceae, which would be consistent with previous study (Yang et al. 2018; Wang et al. 2019), and the Po-α WGD in the *P. oleracea* lineage occurred after its divergence from *P. amilis*. Finally, using a synonymous substitution rate per site per year of 7.54 × 10^−9^ for Caryophyllales, we estimated that the 2 rounds of WGD occurred around 66.30 and 7.47 Mya, respectively (Fig. 2A).

### More gene copies encoding key enzymes/transporters for C_4_ and CAM probably laid the foundation for the coexistence of 2 CCMs in one leaf in *P. oleracea*

Recently, the expression patterns of *P. oleracea* genes encoding some key enzymes involved in C_4_ or CAM metabolism were explored via RNA-seq (Christin et al. 2014; Ferrari et al. 2019; Gilman et al. 2022; Moreno-Villena et al. 2022). Yet, such analysis was still not complete due to the lack of the genome of *P. oleracea*, and thus, the investigation to the mechanisms of the integrated C_4_ and CAM photosynthesis pathways was highly limited. Here, we systematically identified the *P. oleracea* homologs of genes encoding key enzymes/transporters of C_4_ and CAM based on their functional annotation. Notably, most of these genes had a much higher gene copy number in *P. oleracea* than that in other species (Supplemental Table S6). Clustering and phylogenetic analyses showed that WGDs contributed much to the increased gene copy numbers, such as the genes encoding aspartate transaminase (AspAT) (all the 10 genes were from WGD), phosphate dikinase (PPDK) (all the 8 genes were from WGD), and β-carbonic anhydrase (β-CA) (9 of the 11 genes were from WGD) (Supplemental Fig. S8). We then separated these genes into day- or night-phased genes based on their expression pattern in normal and stress conditions, as well as their preferential expression in leaves resulting in a list of 29 C_4_-related and 9 CAM-related genes (Supplemental Figs. S9 and S10). Among the 29 C_4_-related genes, we noticed genes encoding 12 key enzymes or transporters, including β-CA, NAD(P)-ME, PEPC, and AspAT. These gene copies were highly expressed during the day under normal conditions but were downregulated under stress (Supplemental Fig. S9). Similarly, the list of CAM-related copies exhibits higher expression at night under stress. The 9 CAM-related copies encoded 2 β-CAs, 1 PEPC-K, 2 CAM-PEPCs, 2 aluminum-activated malate transporters, 1 PPDK, and 1 PPDK-RE. In contrast to most of the single-copy genes previously identified based on transcriptome data, those newly identified photosynthesis-related gene copies might shed comprehensive light onto future bioengineering that achieves CAM-to-C_4_ progression.

### Analysis of the origin and evolution of PEPC gene copies specific for CAM and C_4_ pathways at the genome-wide scale

**Figure 3.**
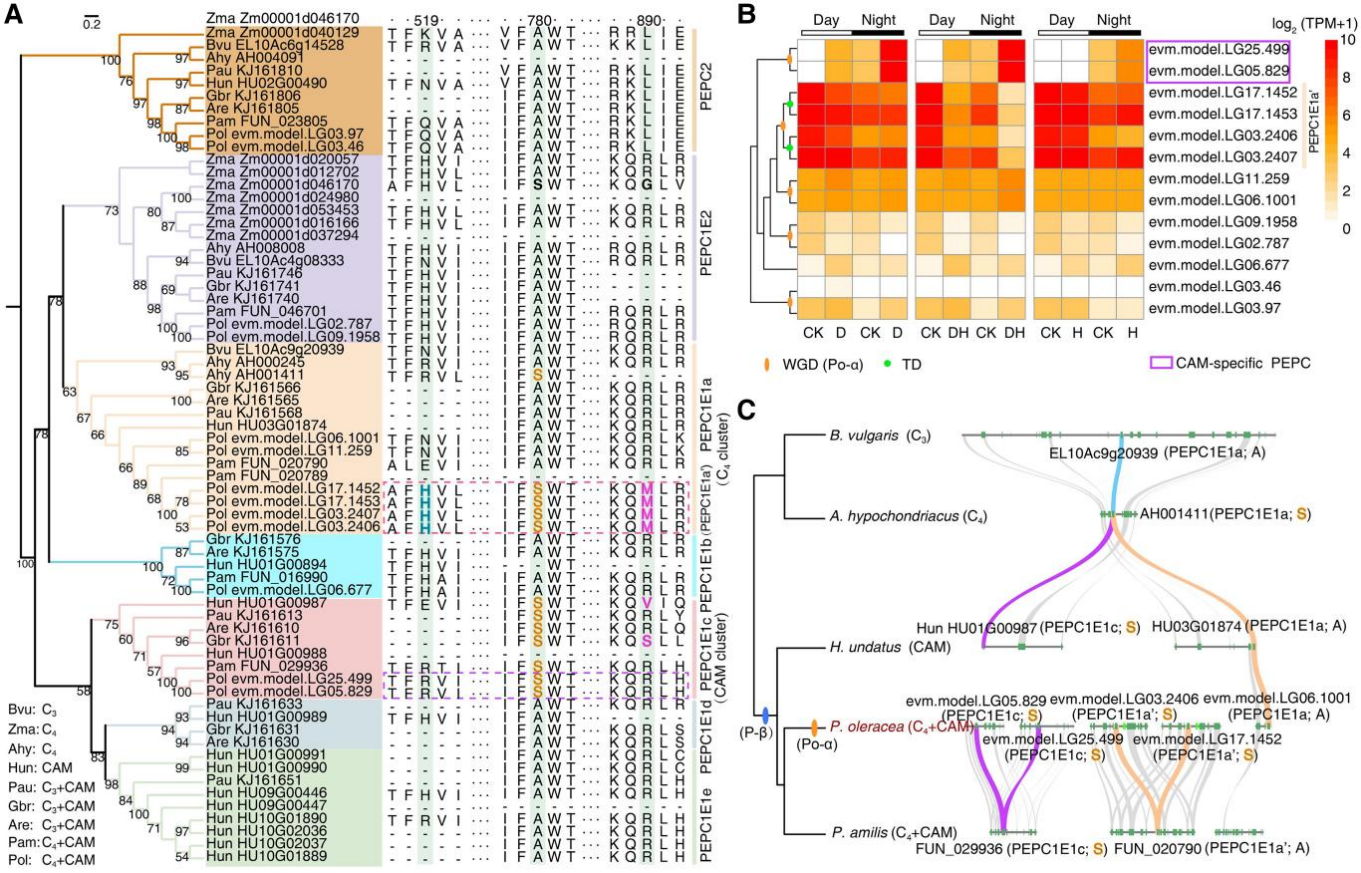
Analysis of C_4_- and CAM-specific PEPC genes in the common purslane genome. **A)** Phylogenetic tree of PEPC proteins from *P. oleracea* (Pol, C_4_ + CAM), *P. amilis* (Pam, C_4_ + CAM), *H. undatus* (Hun, CAM), *A. hypochondriacus* (Ahy, C_4_), *B. vulgaris* (Bvu, C_3_), *Z. mays* (Zma, C_4_), and several identified PEPC proteins from *Pereskia aureiflora* (Pau, C_3_ + CAM), *Anacampseros retusa* (Are, C_3_ + CAM), and *Grahamia bracteata* (Gbr, C_3_ + CAM) in Caryophyllales. Only bootstrap values greater than 50% were shown. Branches were colored according to the PEPC subclass. A multiple sequence alignment of partially conserved amino acids was shown to the right. Sites at positions of 519 (known as position of D509 in the *Kalanchoë* genome), 780, and 890 (according to the numbering in Zm00001d046170) were highlighted in light-green background. Potentially functional C_4_- and CAM-specific PEPC proteins were highlighted in magenta and purple boxes, respectively. **B)** Gene expression of PEPC genes identified in *P. oleracea* under heat and drought treatments during the day and night. Orange ovals in the gene trees to the left represent WGD Po-α events; green dots represent TD events. CAM-specific PEPC genes were highlighted in magenta boxes. **C)** Synteny analysis of C_4_- and CAM-specific PEPC genes in the *P. oleracea*, *P. amilis*, *H. undatus*, *A. hypochondriacus*, and *B. vulgaris* genomes in Caryophyllales. CK, control group; D, drought group; H, heat group; DH, drought combined heat group.

PEPC, as an essential enzyme, can catalyze the fixation of HCO_3_^−^ to the receptor phosphoenolpyruvate resulting in the formation of oxaloacetate during the process of CO_2_ fixation. Prior studies revealed one key CAM-specific PEPC isoform, which was upregulated under drought at night, and one C_4_-specific *PEPC*, which exhibited high expression level during daytime under well-watered condition (Christin et al. 2014; Ferrari et al. 2020b). However, a general atlas of all PEPC gene copies in *P. oleracea* has not been explored. Taking advantage of the whole-genome gene information, we first performed a phylogenetic analysis on PEPC proteins encoded by the *P. oleracea* (C_4_ + CAM), *P. amilis* (C_4_ + CAM) (Gilman et al. 2022), *H. undatus* (CAM) (Chen et al. 2021; Zheng et al. 2021), *B. vulgaris* (C_3_) (Dohm et al. 2013), and *A. hypochondriacus* (C_4_) (Lightfoot et al. 2017) genomes as well as several others (C_3_ + CAM) that have been reported in the Caryophyllales (Christin et al. 2014) (Fig. 3A and Supplemental Table S7). Of the 13 PEPC proteins in *P. oleracea*, 2 were grouped into the PEPC2 cluster, another 2 into PEPC1E2, and 9 into PEPC1E1; this latter cluster consists of 6 PEPC1E1a (putative C_4_-specific subcluster), 1 PEPC1E1b, and 2 PEPC1E1c (putative CAM-specific subcluster) proteins. After analyzing the expression level of each PEPC gene, we obtained 4 C_4_-specific *PoPEPC1E1a′* genes with high expression level during the day regardless of growth conditions and 2 CAM-specific *PoPEPC1E1c* genes being highly expressed under stress conditions at night (Fig. 3B). Strikingly, the expression levels of the 2 CAM-specific genes were hundreds to thousands of times higher under stress at night than in the control samples or stress samples during the day (Supplemental Fig. S11A) and was demonstrated by reverse transcription quantitative PCR (RT-qPCR) (Supplemental Figs. S11B and S12).

According to previous studies, C_4_/CAM-specific PEPC proteins that are functional or exhibit high efficiency during photosynthesis generally harbor a serine (S) at residue 780 (using the amino acid numbering of the protein encoded by Zm00001d046170 in maize [*Zea mays*]), while other PEPC proteins carry a conserved alanine (A) at the equivalent position (Svensson et al. 2003; Christin et al. 2014). Although the above observation was not absolute (Rao et al. 2008; Christin et al. 2014), we determined that it likely holds true in *P. oleracea* (Fig. 3A). Indeed, protein sequence alignments with other PEPCs from classical C_4_ and CAM species highlighted 4 PEPC1E1a proteins in *P. oleracea* (PoPEPC1E1a) with an S residue at position 780 (classified as PoPEPC1E1a′; the other 2 had an A at this position), as well as 2 PoPEPC1E1c enzymes with S at position 780 (Fig. 3A). Notably, the other reported C_4_ + CAM plant *P. amilis* had only one copy each for the PEPC1E1a and PEPC1E1c subclusters, of which only PaPEPC1E1c carried the S residue. We also examined residue 890 (according to the numbering in Zm00001d046170), where an arginine (R) in PEPC was shown to support tight inhibitor binding in C_3_ plants, while C_4_-related PEPCs have a glycine (G) or other residues with lower inhibitor affinity (Paulus et al. 2013). All 4 candidate C_4_-specific PoPEPC1E1a′ proteins possessed a methionine (M) instead of an R at this position, which may be responsible for the high efficiency of photosynthetic carbon fixation in *P. oleracea*. By contrast, position 890 in the only PEPC1E1a of *P. amilis* harbored an R, like most C_3_ plants. We also detected residue 519 (according to the numbering in Zm00001d046170) known as position of D509; an aspartic acid (D) residue was demonstrated to reduce malate inhibition in the *Kalanchoë* genome (Yang et al. 2017). Histidine (H) and glutamic acid (E) were present at this position in PEPC1E1a′ in the *P. oleracea* and *P. amilis* genomes respectively, which was consistent with the result in the paper of the *P. amili*s genome (Gilman et al. 2022). Overall, these results provided further evidence at the molecular level that *P. oleracea* is a C_4_ and CAM plant and that the 4 C_4_-specific *PoPEPC1E1a′* and 2 *PoPEPC1E1c* genes we identified here may be keys to its C_4_ and CAM photosynthesis.

In addition, to explore the origin and evolution of PEPC genes in *P. oleracea*, we compared the genomes among *P. oleracea* (C_4_ + CAM), *P. amilis* (C_4_ + CAM), *H. undatus* (CAM), *A. hypochondriacus* (C_4_), and *B. vulgaris* (C_3_) in the Caryophyllales. Synteny analyses of *PEPC1E1a* and *PEPC1E1c* genes in these species suggested that the P-β WGD event may produce the ancestral copies of C_4_ and CAM-specific PEPC genes (Fig. 3C). Combing with the information of phylogenetic trees and collinearity evidence (Fig. 3, A and C), we speculated that the ancestral *PEPC1E1a’* copies in *P. oleracea* genome might be duplicated from the *PEPC1E1a* copy, and then 4 copies were generated by the recent Po-α WGD and TD events (Fig. 3, A and B). By contrast, the ancestral copy of 2 *PEPC1E1c* copies probably came from the P-β WGD event, followed by duplication after the Po-α WGD (Fig. 3). Overall, P-β WGD, Po-α WGD, and TD events contributed to the occurrence of multicopies of C_4_- and CAM-specific PEPC genes in *P. oleracea*.

### Gene duplications produced functionally differentiated β-CA genes involved in C_4_ + CAM pathways

**Figure 4.**
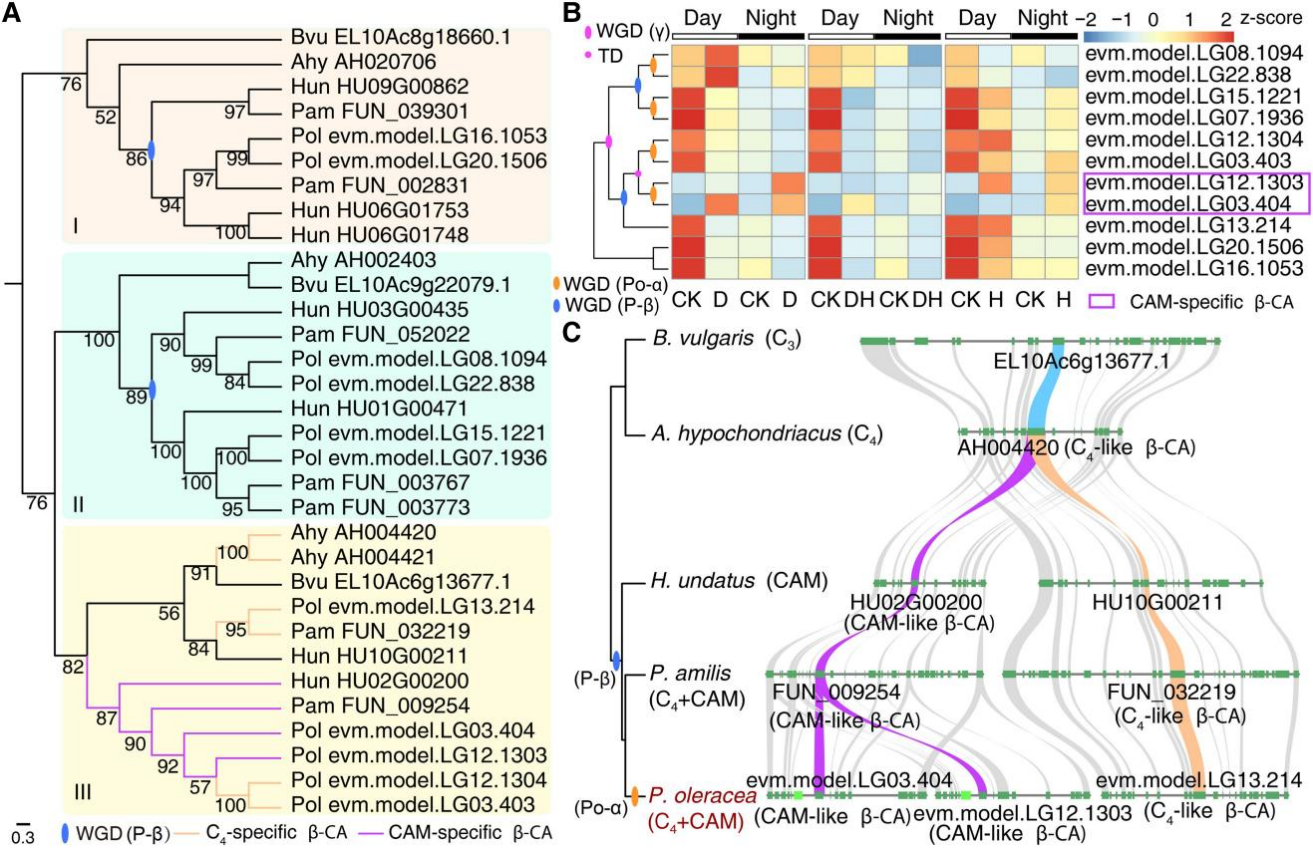
Origin and evolution of C_4_- and CAM-specific β-CA genes in Caryophyllales. **A)** Phylogenetic tree of β-CA genes from *P. oleracea* (Pol), *P. amilis* (Pam), *H. undatus* (Hun), *A. hypochondriacus* (Ahy), and *B. vulgaris* (Bvu) genomes in Caryophyllales. **B)** Gene expression of β-CA genes identified in *P. oleracea* under heat and drought treatments during the day and night. Magenta, blue, and orange ovals in the gene trees to the left represent γ, P-β, and Po-α WGD events, respectively; magenta dots represent TD events. CAM-specific β-CA genes were highlighted in magenta boxes. **C)** Synteny analysis of C_4_- and CAM-specific β-CA genes (clade III in Fig. 4A) in the *P. oleracea*, *P. amilis*, *H. undatus*, *A. hypochondriacus*, and *B. vulgaris* genomes in Caryophyllales. CK, control group; D, drought group; H, heat group; DH, drought combined heat group.

In both CCMs pathways, CO_2_ is converted to HCO_3_^−^ by a key enzyme β-CA, which has been identified and classified into C_4_- and CAM-specific β-CA based on transcriptome data in *P. oleracea* (Ferrari et al. 2019). To explore the origin and evolutionary process of C_4_ and CAM pathway in *Portulaca* more comprehensively and accurately, we analyzed evolution of β-CA genes coding first-step key enzymes in C_4_ and CAM pathways in the genomes of *P. oleracea* (C_4_ + CAM), *P. amilis* (C_4_ + CAM), *H. undatus* (CAM), *A. hypochondriacus* (C_4_), and *B. vulgaris* (C_3_) in the Caryophyllales. Firstly, we identified 32 β-CA genes in the above 5 species including 11 in *P. oleracea*, 7 in *P. amilis*, 7 in *H. undatus*, 4 in *A. hypochondriacus*, and 3 in *B. vulgaris* (Fig. 4A). By constructing phylogenetic trees, we have divided these genes into 3 categories, of which clade III contained C_4_- (*FUN_032219*) and CAM-specific (*FUN_009254*) genes in *P. amilis* (Gilman et al. 2022) and the corresponding 3 C_4_-specific and 2 CAM-specific β-CA gene copies in *P. oleracea* (Fig. 4B). Synteny analyses of these genes indicated that P-β and Po-α WGD events contribute to the occurrence of multicopies of β-CA genes in *P. oleracea* (Fig. 4, B and C). Furthermore, collinearity analysis of the chromosomal segment where C_4_- and CAM-specific genes located showed that P-β WGD event produces C_4_-like cluster (HU10G00211 in *H. undatus*, FUN_032219 in *P. amilis* and evm.model.LG13.214 in *P. oleracea*) and CAM-like cluster (HU02G00200 in *H. undatus*, FUN_009254 in *P. amilis* and evm.model.LG03.404 and evm.model.LG12.1303 in *P. oleracea*), which indicated that P-β WGD event resulted in the origin of 2 types of *β-CA* genes (Fig. 4C). Interestingly, TD events prior to Po-α WGD event in *P. oleracea* genome led to repeated evolution of C_4_-specific β-CA genes, while Po-α WGD event occurring independently in *P. oleracea* may promote the photosynthetic efficiency by increasing the gene expression dosage (Fig. 4, B and C). Similar to the evolutionary pattern of *PEPC* gene, but more clearly, duplication preceded functional evolution: P-β WGD event produced 2 ancestral genes of functionally differentiated (C_4_- and CAM-specific) β-CA genes involved in C_4_ + CAM pathways.

### Facultative CAM likely recruits a set of cis DNA element

**Figure 5.**
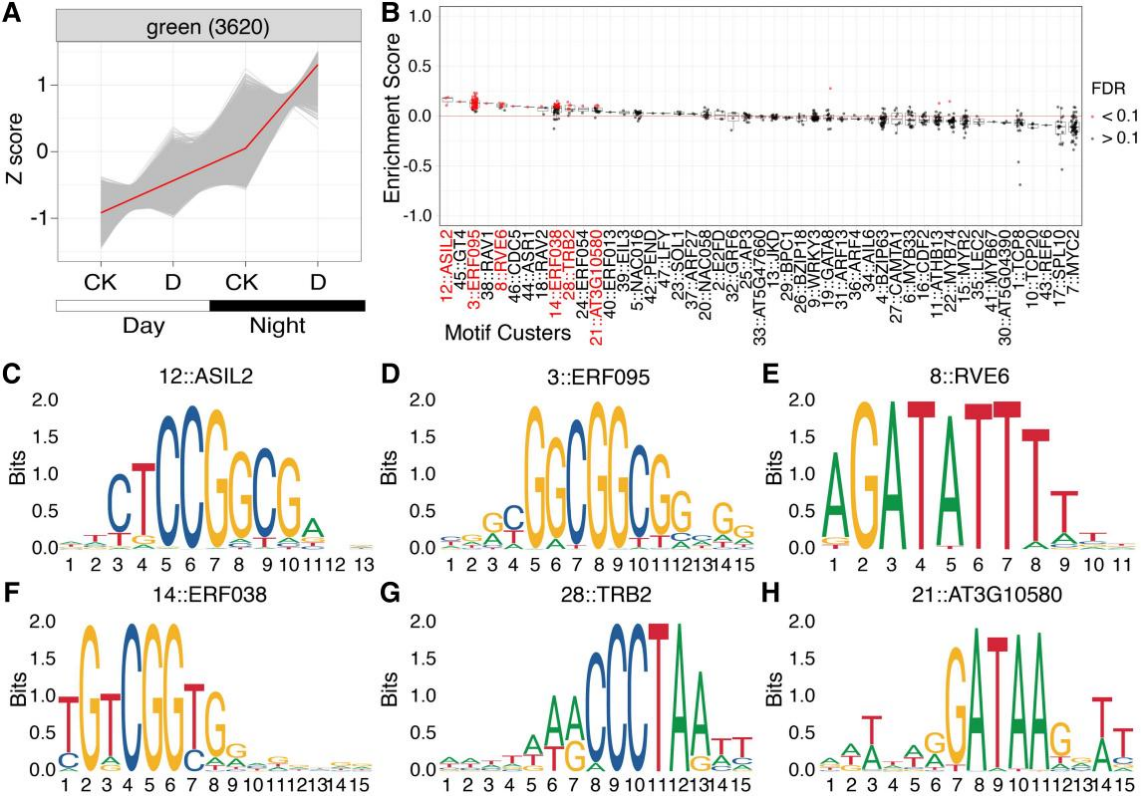
CAM-specific module is enriched with motifs from clusters 12, 3, 8, 14, 28, and 21. **A)** Gene expression pattern of CAM-specific green module containing 3,620 genes displays more upregulated gene expression after drought treatment at nighttime; data were presented as Z-score; red line connects the mean of expression in each sample. **B)** Enriched TF binding sites placed in motif clusters; red dots indicate significantly enriched motifs (FDR < 0.1) compared with genomic background; motif clusters with more than 3 enriched motifs were highlighted in red text. **C** to **H**), Sequence logo of representative motifs from motif clusters 12, 3, 8, 14, 28, and 21.

Patterns of gene expression were largely determined by interactions between cis- and trans-factors. To investigate the recruitment of cis-elements by facultative CAM associated genes, we first clustered genes using Weighted correlation network analysis (WGCNA) and identified 27 modules with distinct expression pattern (Supplemental Fig. S13). Day- or night-specific modules were defined by a lack of response to drought but preferential expression at day or night (Supplemental Figs. S14A and S14C). Two modules were defined as drought specific due to the strong induction of gene expression by drought at both day and night (Supplemental Figs. S14E and S14G). A module containing both CAM-specific *PEPC1E1c′* (*evm.TU.LG05.829*, *evm.TU.LG25.499*) and β-CA (*evm.TU.LG12.1303*) was recognized as CAM specific and exhibited enhanced upregulation of expression at night under drought condition (Fig. 5A).

Find Individual Motif Occurrences (FIMO), a software designed for scanning DNA or protein sequences with given motifs (Bailey et al. 2015), was used to scan known transcription factor (TF) binding sites from JASPAR database (656 motifs from 47 motif clusters) in upstream 2,000-bp promoter sequences, and the frequency of occurrence in each module was compared with genomic background. This identified motifs from clusters 7 (MYC2), 4 (bZIP63), 15 (MYR20), and 1 (TCP8) that were significantly enriched in genes preferentially expressed in the day (Supplemental Fig. S14B). Cluster 8 (RVE6) was enriched in genes preferentially expressed in the night, and 2 drought-specific modules were enriched with clusters 21 (AT3G10580), 7 (MYC2), and 4 (bZIP63) motifs (Supplemental Fig. S14, D, F, and H). There were few overlapping motifs between the CAM-specific module and night- or drought-specific modules except for an evening element cluster 8 (RVE6) and cluster 21 (AT3G10580); most enriched motifs belong to clusters 12 (ASIL2), 3 (ERF095), 14 (ERF038), and 28 (TRB2) (Fig. 5, B to H). Consistent with this analysis of enriched cis-elements, cognate TF genes such as *REVEILLE 6* (*RVE6*, evm.TU.LG06.1912) and 2 *TELOMERE REPEAT BINDING FACTOR 2* (*TRB2*) gens (evm.TU.LG13.1168 and evm.TU.LG21.800) as well as ethylene-response factor (ERF) TF genes including *DREB2A/2B* (evm.TU.LG19.575) and 3 cytokinin response factor genes (evm.TU.LG03.1986, evm.TU.LG04.3303, evm.TU.LG14.283) were also identified in CAM-specific module (Supplemental Tables S8 to S11). Taken together, these data suggest that facultative CAM likely results from the recruitment of a CAM-specific regulatory network using a specific set of cis-elements that are likely absent in night- or drought-specific network.

### Gene duplications may contribute to common purslane stress resistance

**Figure 6.**
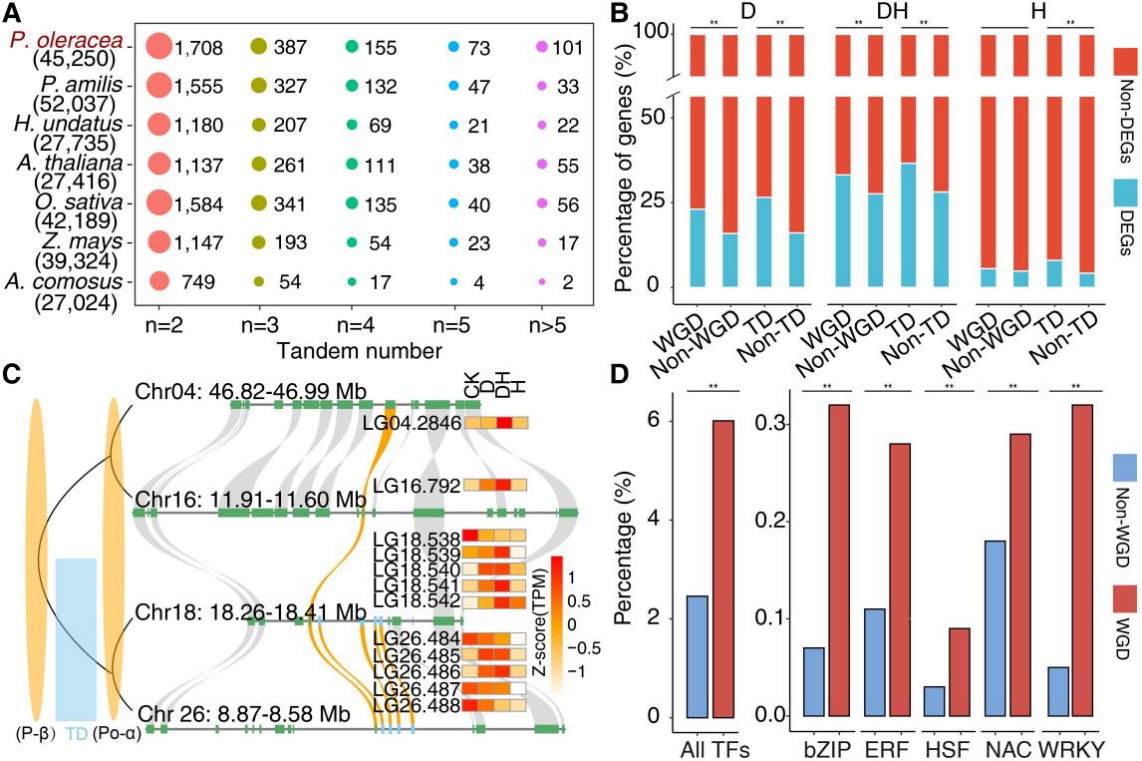
Analyses of WGD and TD of the common purslane genome. **A)** Comparison of the number of tandem duplicates in different species. **B)** Percentage of DEGs and non-DEGs for WGD and non-WGD, TD, and non-TD genes upon heat and/or drought treatment. **Significant difference by *P* < 0.01 (chi-square test). **C)** An example of gene expansion from WGD and TD events in the *P. oleracea* genome. The genes encoding ELIPs, thought to act as photoprotectants, are highly upregulated upon heat and drought treatments. The highlighted orange regions are from WGD, while the light blue ones are from TD. **D**) TFs, especially several stress-related TF families, are significantly enriched in the WGD gene set compared with the non-WGD set. **P < 0.01 by chi-square test. CK, RNA-seq data under normal conditions; D, drought; H, heat; and DH, combined drought and heat.

The expansion and contraction of gene families have a profound influence in shaping stress resistance and driving phenotypic diversity and adaptive evolution in flowering plants (Jiao et al. 2011; Chen et al. 2013; Wu et al. 2020; Zhang et al. 2020; Wang et al. 2022). We identified 32,605 putative orthologous gene clusters composed of 475,125 protein-coding genes from 528,054 genes across 16 plant species used in this study. Compared with the other 15 species, the *P. oleracea* genome possessed the largest proportion, as well as the most gene cluster expansions (9,375 gene clusters expanded) (Fig. 2A and Supplemental Table S12). The *P. oleracea* genome also exhibited the largest number of species-specific gene clusters (1,605) compared with its relative species (*B. vulgaris*, *S. oleracea*, and *H. undatus*) (Supplemental Fig. S15). Moreover, we detected 7,740 genes derived from WGD by self-synteny analysis, as they retained at least 3 copies after 2 rounds of WGD events, as well as 6,330 genes derived from TDs by Basic Local Alignment Search Tool of Protein (BLASTP) and chromosomal location analysis. We also observed that the number of tandem duplicates in *P. oleracea* is higher than that in other species (Fig. 6A). GO analysis revealed that WGD genes are highly enriched for the “biological regulation” and “DNA binding” categories, while TD genes tended to be enriched for the “enzyme inhibitor activity” and “response to stress” categories (Supplemental Fig. S16A). In addition, among the differentially expressed genes (DEGs) under drought and/or heat treatment, WGD and TD genes account for more than 30% (Supplemental Fig. S16B). To assess the consequence of these WGD and TD events on gene expression, we conducted an RNA-seq analysis of *P. oleracea* plants grown under control conditions or exposed to heat and/or drought stress. We identified more DEGs among genes having experienced WGD and TD under drought and/or heat treatment compared to non-WGD and nontandem-duplicated genes (Fig. 6B). For example, all 12 copies of a gene encoding early light-induced protein (ELIP), which underwent 2 rounds of WGD and TD events, were differentially expressed in *P. oleracea* seedlings exposed to heat and drought (Fig. 6C).

In addition, we determined the mean relative size of gene families that had been described previously by the OneKP Project (One Thousand Plant Transcriptomes Initiative 2019) in the selected genomes above. Notably, we found that most of these gene families, such as *basic helix-loop-helix* (*bHLH*), *MYB*, and *WRKY*, have more members in the *P. oleracea* genome than in other species (Supplemental Fig. S17). Further analysis revealed that TF genes are significantly enriched in the WGD gene set, including several reported stress-related TF gene families, such as *heat shock factor* (*HSF*), *basic Leucine zipper* (*bZIP*), *NAC*, *WRKY*, and *ERF*, and other important families (Fig. 6D and Supplemental Fig. S18). By contrast, we detected fewer TF genes in the TD list (Supplemental Fig. S19). For instance, a member from the *HSF A* (*HsfA*) family was present in 5 copies in the genome from TDs and WGDs; importantly, all 5 copies were highly expressed upon drought and heat stress (Supplemental Fig. S20). These results suggested that the high frequency of WGD and TD genes in the *P. oleracea* genome may have contributed to its adaptation to environmental stress.

## Discussion

*Portulaca* is the only genus in the Portulacaceae family, with substantial variation in chromosome number between species (Danin et al. 1978; Ocampo and Columbus 2012; Walter et al. 2015). The base chromosome number of *Portulaca* was inferred to be *x* = 9 (Ocampo and Columbus 2012). Common purslane had been classified into several subspecies or microspecies (and as such is referred to as a *P. oleracea* complex) with somatic chromosome number of 2*n* = 18, 36, 52, and 54 in Chromosome Counts Database (Rice et al. 2015). Notably, the FISH experiment, *k*-mer, and genomic analysis indicated that the common purslane we used is diploid with a chromosome number of 2*n* = 52. There could be 2 possibilities to explain the distinct chromosome number: one is that the accession we sequenced may have been a base chromosome change prior to the Po-α WGD, and the other is that post-WGD rearrangement led to the current number (2*n* = 52) of chromosomes.

Plants from the Portulacaceae were reported to possess diverse photosynthesis pathways such as C_4_ + CAM and C_3_-C_4_ + CAM (Koch and Kennedy 1980; Voznesenskaya et al. 2010, 2017; Ferrari et al. 2020b). More than 10 common purslane genotypes were recently demonstrated to perform C_4_ + CAM (Ferrari et al. 2020b), making common purslane the most widely studied species for its potential as a C_4_/CAM model (Ferrari et al. 2020a). Our research reported a high-quality, chromosome-level genome of common purslane with 2 rounds of WGD occurring around 66.30 and 7.74 Mya, respectively. Our analysis of the common purslane genome revealed that most genes are present in multiple copies, including many that encode key enzymes or transporters involved in photosynthesis. We successfully assigned several PEPCs to C_4_-specific (4 enzymes) and CAM-specific (2 enzymes) groups with amino acids previously associated with these photosynthetic pathways, lending further support that common purslane is a C_4_ + CAM plant. Importantly, although C_4_- and CAM-related genes were previously reported in the related species Paraguayan purslane, such as *PaPPC-1E1a′* (FUN_020790, named *PaPEPC1E1a* in this study) and *PaPPC-1E1c* (FUN_029936, named *PaPEPC1E1c* in this study) (Gilman et al. 2022), *PaPEPC1E1a* encoded an enzyme with the derived amino acids (E519 and A780) that have previously not been associated with either CAM or C_4_ photosynthesis. In addition, PaPEPC1E1a harbored the residue (R890) for tight inhibitor binding, much like PEPC1E1a from CAM-type dragon fruit. Further work will be required to understand the impact of these amino acids on the efficiency of PEPC activity and C_4_ photosynthesis in Paraguayan purslane.

Land plants have evolved C_4_ and/or CAM photosynthesis well over 100 times to adapt to stressful environments such as low CO_2_, high temperatures, and drought (Edwards 2019). Although C_4_ and CAM evolutionary trajectories are largely distinct, *Portulaca* demonstrates that they also can be compatible (Edwards 2019; Hibberd 2022; Moreno-Villena et al. 2022). A de novo genome assembly for *P. amilis* was reported recently. It was helpful to reveal coexpression networks that may support C_4_-CAM compatibility (Gilman et al. 2022). Our analysis of the common purslane genome focused more on the evolutionary contribution of gene duplication to C_4_-CAM compatibility. We explored this important issue through the origin and evolution of 2 key enzymes: PEPC and β-CA. Since PEPC seems to have gone through a more complex process in the evolutionary history, β-CA may be more informative than PEPC in explaining the origin and evolution of the C_4_ and CAM pathways. Phylogenetic trees and synteny analyses indicated that the P-β WGD produced functionally differentiated β-CA genes that become involved in the C_4_ + CAM pathways. However, there could be multiple possible histories of C_4_ related-gene evolution if the effects of convergent evolution on gene tree topology are taken into account. Interestingly, TD events prior to Po-α WGD event in common purslane genome led to repeated evolution of C_4_-specific β-CA genes, which provided further evidence for that C_4_ photosynthesis has repeatedly evolved in plants.

Upstream regulatory sequences play a critical role in determining spatial temporal gene expression. The common purslane genomic sequence provides an excellent opportunity to initiate an understanding of the cis-regulation of facultative CAM. Cis-element enrichment analysis identified G-box (7:MYC2) and TCP (1:TCP8) in day-specific genes and an evening element (8:RVE6) in night-specific genes, consistent with the involvement of these elements in diel regulation of gene expression (Wang et al. 2019). Drought-specific genes were enriched with clusters 7 (MYC2) and 4 (bZIP63), which coincide with JA or ABA associated drought response elements such as G-boxes or ABREs (Liu et al. 2018; Soma et al. 2021). It has been hypothesized that facultative CAM likely resulted from combinatorial rewiring of circadian and drought responsive networks (Gilman et al. 2022). Indeed, we discovered that the promoters of CAM-specific genes in common purslane are significantly enriched with evening-specific elements and drought-response-related cis-elements. Both the evening element RVE6 and the midnight element Telobox (TRB2, which is identical to motif cluster 28) are overrepresented in CAM-specific promoters, and this is consistent with CAM cycling genes in the C_3_ facultative CAM species *Sedum album* (Wai et al. 2019). However, *S. album* utilizes ABRE or ABRE-like motifs from ABA-dependent drought regulatory networks, while common purslane recruited a different set of drought-related motifs known as ERF motifs recognized by Dehydration responsive element binding protein (DREB)/ERF TFs and linked with ABA-independent drought regulatory networks. This suggests that the utilization of various drought response regulatory networks along with the conserved recruitment of evening-specific elements could be a possible mechanism for CAM-specific gene expression. Overall, these data shed light on the convergent evolution of facultative CAM and provide potential regulatory modules for incorporating C_4_/CAM into crops in the future.

Climate change has far-reaching and adverse effects on crop yields and human nutrition. To make matters worse, an increasing world population will require that current food production to be doubled by the year 2050. Responding to these problems will require the development of high-yield, as well as stress-tolerant crops. To achieve this, there has been an ongoing global initiative to engineer C_4_ or CAM pathways into C_3_ crops or the CAM pathway into C_4_ plants like maize. A key step toward engineering C_4_ rice was achieved through constitutive expression of maize *GOLDEN2-LIKE* genes, and field-grown transgenic plants resulted in a 30% to 40% increase in both vegetative biomass and grain yield (Wang et al. 2017; Li et al. 2020). Here, we accurately identified all gene copies of the key proteins involved in the C_4_ + CAM photosynthetic pathway. Further analysis of their diurnal expression patterns, as well as their key functional sites will allow the identification of important gene copies as the targets for engineering crops with the C_4_ and/or CAM photosynthetic pathway. For example, based on series analysis for PEPC gene copies, we would select evm.model.LG25.499, evm.model.LG05.829, evm.model.LG17.1452, evm.model.LG17.1453, evm.model.LG03.2406, and evm.model.LG03.2407 as the potential targets from 13 PEPC gene copies. Overall, we believe that this study will be an invaluable resource to investigate the integration of different photosynthetic pathways. Our comprehensive and in-depth analysis of a genome underpinning this complex photosynthetic pathway could provide potential targets for C_4_ + CAM engineering.

## Materials and methods

### Plant materials and DNA sequencing

In this work, we used wild common purslane (*P. oleracea*), which grows widely in Beijing, China. These plants were grown in an environmental chamber (10 h day/14 h night). Leaves from a 30-day-old mature plant were harvested and frozen immediately in liquid nitrogen. Genomic DNA was extracted using a QIAGEN Genomic Kit and used as material to construct sequencing libraries following the standard protocols of Oxford Nanopore Technologies. Briefly, DNA was size-selected using a BluePippin instrument (Sage Science, USA) before end-repair and adaptor ligation on the resulting blunt ends. Finally, libraries were sequenced on a PromethION platform. DNA from the same batch of samples was used to prepare Illumina libraries, following the standard manufacturer's protocol (Illumina). Libraries were sequenced on an Illumina Novaseq 6000 platform as 150-bp pair-end reads.

To assess the response of common purslane to stress, 1-mo-old plants were separated into 4 experimental groups, each being subjected to different conditions for 7 days: (i) controls in normal environment conditions (14-h-light/10-h-dark photoperiod [with lights on from 6 AM to 8 PM], with a 28 °C/22 °C day/night temperature cycle), (ii) drought stress (no water irrigation for 7 days), (iii) heat treatment (water applied regularly and temperature raised to 45 °C during the day for the last 4 days), and (iv) combined heat and drought stress (no water irrigation for 7 days and 45 °C during the day for the last 4 days). Leaves were collected after 7 days of treatment during the day (10 AM) and at night (12 AM), with 3 biological replicates.

### Malate measure

After the sample was thawed and smashed, an amount of 0.05 g of the sample was mixed with 500 µL of 70% (v/v) methanol/water. The sample was vortexed for 3 min under the condition of 2,500 r/min and centrifuged at 12,000 r/min for 10 min at 4 °C. Then, 300 *μ*L of supernatant was placed into a new centrifuge tube and into a −20 °C refrigerator for 30 min. Then, the supernatant was centrifuged again at 12,000 r/min for 10 min at 4 °C. After centrifugation, 200 *μ*L of supernatant was transferred for further LC-MS analysis.

The sample extracts were analyzed using an LC-ESI-MS/MS system. The analytical conditions were as follows: HPLC column, ACQUITY HSS T3 (i.d. 2.1 × 100 mm, 1.8 *μ*m); solvent system, water with 0.05% (v/v) formic acid (A), acetonitrile with 0.05% formic acid (v/v) (B); gradient started at 5% B (0 min), increased to 95% B (8 to 9.5 min), and finally ramped back to 5% B (9.6 to 12 min); flow rate, 0.35 mL/min; temperature, 40 °C; and injection volume, 2 *μ*L.

### BioNano optical maps and Hi-C sequencing

For BioNano physical mapping, DNA extracted from leaves were subject to manufacturer recommended protocols for library preparation (Bionano PrepTM Animal Tissue DNA Isolation Kit [CAT#80002]/Bionano PrepTM Plant DNA Isolation Kit [CAT#80003]) and optical scanning provided by BioNano Genomics (https://bionanogenomics.com), with the labeling enzyme Direct Label Enzyme (DLE) (Bionano PrepDLS Labeling DNA Kit, CAT#80005). Labeled DNA samples were loaded and run on the Saphyr system (BioNano Genomics) in Grandomics.

To anchor hybrid scaffolds onto the chromosome, genomic DNA was extracted for the Hi-C library from samples. Then, we constructed the Hi-C library and obtained sequencing data via the Illumina Novaseq/MGI-2000 platform. In brief, freshly harvested leaves were cut into 2 cm pieces and vacuum infiltrated in nuclei isolation buffer supplemented with 2% (v/v) formaldehyde. Crosslinking was stopped by adding glycine and additional vacuum infiltration. Fixed tissue was then ground to powder before resuspending in nuclei isolation buffer to obtain a suspension of nuclei. The purified nuclei were digested with 100 units of DpnII and marked by incubating with biotin-14-dATP. Biotin-14-dATP from nonligated DNA ends was removed owing to the exonuclease activity of T4 DNA polymerase. The ligated DNA was sheared into 300 to 600 bp fragments and then was blunt-end repaired and A-tailed, followed by purification through biotin-streptavidin–mediated pull down. Finally, the Hi-C libraries were quantified and sequenced using the Illumina Novaseq/MGI-2000 platform.

### Transcriptome sequencing

Total RNA was isolated from higher leaves, lower leaves, higher stems, lower stems, roots, immature flowers, and mature flowers of common purslane using TRIzol reagent. mRNA-seq libraries were constructed using a TruSeq RNA Library Preparation Kit (Illumina, USA) following the manufacturer's recommendations, and 150-bp paired-end sequencing was performed on a Novaseq 6000 platform to assist gene prediction. Samples collected during the stress treatment experiments described above were also subjected to total RNA extraction using a Promega ReliaPrep RNA Tissue Miniprep System Kit. Sequencing libraries were then prepared and sequenced as above.

### Preparation of chromosome spreads and FISH

Preparation of chromosome spreads and FISH were performed according to Huang et al. (2021). Briefly, 1-cm root tip segments were pretreated in 0.05% (w/v) 8-hydroxyquinoline for 2 h at 25 °C, fixed in 3:1 ethanol:acetic acid (v/v) fixative overnight, and kept at −20 °C until use. An enzymatic solution consisting of 2% (w/v) cellulase and 1% (w/v) pectolyase was used to digest the root tips at 37 °C for 3 h. Then, 10 µL of 1:3 ice-cold acetic acid:methanol mixture (v/v) was added, and the root tips were broken with tweezers, mounted onto a glass slide and allowed to air-dry. FISH was performed using a hybridization mixture (10 µL) containing 50% (w/v) formamide, 10% (w/v) dextran sulfate in 2× SSC (saline sodium citrate), and 40 ng of biotin-labeled 45S rDNA and digoxigenin-labeled 5S rDNA probes. Hybridization was carried out for 16 h at 37 °C. Digoxigenin-labeled and biotin-labeled probes were detected using rhodamine-conjugated antidigoxigenin and fluorescein-conjugated avidin, respectively. Chromosomes were counterstained with DAPI (4′,6-diamidino-2-phenylindole) in antifade solution (Vector Laboratories, USA) under a coverslip. The slides were examined with an Axio Imager Z.2 Zeiss microscope (Zeiss, Oberkochen, Germany) equipped with a Cool Cube 1 camera (Metasystems, Altlussheim, Germany) and appropriate optical filters. Final image adjustments were performed with Adobe Photoshop CC.

### Genome assembly and quality assessment

The raw Nanopore data were corrected by NextDenovo software (seed_cutoff = 25k; reads_cutoff = 1k) (https://github.com/Nextomics/NextDenovo). The corrected reads were then assembled using smartdenovo software (-k 21, -j 3,000) (https://github.com/ruanjue/smartdenovo) to obtain contigs for the preliminary assembled genome. Contig sequences were polished with the Nanopore reads and Illumina reads and used as input for the Nextpolish software (default) (Hu et al. 2020). BioNano data adopt single-enzyme digestion technology, with the DLE-1 enzyme used to digest genomic DNA to obtain raw data. We constructed longer super-scaffolds by anchoring the polished contig assembly to the BioNano optical map. Then, unique Hi-C read pairs were identified through alignment to the scaffolds by bowtie2 (−very-sensitive -L 30) (Langmead and Salzberg 2012). The DpnII restriction sites were identified along the scaffolds, and the Hi-C interaction signal intensity was used to assign each read to different scaffolds. Finally, the scaffold sequences were clustered into 26 pseudo-chromosome groups by agglomerative hierarchical clustering (bottom-up hierarchical clustering) using LACHESIS software (Burton et al. 2013).

The quality and completeness of the common purslane genome assembly were assessed from 3 aspects. First, the mapping rates of the clean reads obtained from the transcriptomes and genomic DNA were mapped back to the genome assembly by Hisat2 (Kim et al. 2019) and BWA-MEM (Li 2013) with default parameters. Second, the BUSCO score was determined for all predicted genes in the final assembly against the gene list for Embryophyta_odb10 (Simão et al. 2015; Manni et al. 2021). Third, the LAI was employed to infer assembly continuity with default parameters (Ou et al. 2018). Finally, we used Merqury (Rhie et al. 2020) software to estim-ate the consensus QV of the assembly.

### Gene prediction and functional annotation

Gene structure predictions adopted a combination of de novo prediction (Augustus) (Hoff and Stanke 2018), homology prediction (GeMoMa) (Keilwagen et al. 2019), and transcriptome prediction (PASA) (Xu et al. 2006). All 3 approaches were integrated by EvidenceModeler software (Haas et al. 2008). TransposonPSI (http://transposonpsi.sourceforge.net) was used to align and remove genes containing TEs to obtain the final structural genome annotation.

### Repeat annotation and TE analyses

The repetitive sequences were identified using a combination of repeat homology searches and ab initio prediction. For homology searches, Repbase (2018) (Bao et al. 2015) was employed to search the genome using RepeatMasker (Tarailo-Graovac and Chen 2009) with default parameters. For ab initio predictions, a consensus sequence library was built using RepeatModeler (http://repeatmasker.org/RepeatModeler/) with the parameter “-engine ncbi”. Then, LTR_harvest (Ellinghaus et al. 2008), LTR_finder (Xu and Wang 2007), and LTR_retriever (Ou and Jiang 2018) were used to build an LTR library with default parameters. Both libraries were then used for annotating the genome using RepeatMasker, and the detected TEs were combined to obtain the final TE annotation.

### Transcriptome analyses

RNA-seq raw reads were processed using Trimmomatic (Bolger et al. 2014) to remove adaptor sequences and low-quality reads. The clean reads were then mapped to the reference genome using HISAT2 (Kim et al. 2019) with default parameters. The expression abundance values were calculated using Stringtie (Pertea et al. 2016), and we averaged the abundance values from the 3 biological replicates of each sample to obtain levels of gene expression. Finally, we performed differential expression analysis between the corresponding samples by DESeq2 (Love et al. 2014).

### Gene family inference and phylogenomic analysis

The nucleotide and amino acid sequences of 15 representative plant species were downloaded from various sources: Paraguayan purslane (*P. amilis*), amaranth (*A. hypochondriacus*), spinach (*S. oleracea*), sugar beet (*B. vulgaris*), tomato (*Solanum lycopersicum)*, *Arabidopsis thaliana*, *Populus trichocarpa*, grape (*Vitis vinifera*), *Aquilegia coerulea*, pineapple (*Ananas comosus*), maize (*Z. mays*), rice (*Oryza sativa*), *Sorghum bicolor*, and *Amborella trichopoda* from Phytozome (https://phytozome-next.jgi.doe.gov/), and dragon fruit (*H. undatus*) from the Pitaya Genome Database (http://www.pitayagenomic.com/). Gene clusters of putative gene families for these species and common purslane were identified by OrthoFinder (v2.4.0) (Emms and Kelly 2019). Venn diagrams of the selected taxa were generated using Venn diagram (Chen and Boutros 2011). MAFFT (v7.471) (Katoh et al. 2002) was used to align 58 single-copy putatively orthologous gene families. Poorly aligned regions were removed using trimAL (v1.4) (Capella-Gutierrez et al. 2009) with default parameters. The concatenated amino acid alignments were used to construct a species tree by the maximum likelihood method in RAxML (v8.2.12) (Stamatakis 2014) under the “PROTGAMMAAUTO” model with 100 bootstrap replicates. Divergence time for each tree node was inferred using r8s (v1.81) (Sanderson 2003) and gene family expansion and contraction by CAFE (v4.2.1) (De Bie et al. 2006).

### Genomic synteny analyses

Synteny searches were performed to identify syntenic blocks within common purslane and between common purslane and dragon fruit using MCScanX (Wang et al. 2012) by default parameter settings. Dotplots and macrosynteny patterns were drawn by JCVI (https://github.com/tanghaibao/jcvi) and R scripts, respectively.

### Synonymous substitution (*K*_s_) analysis

For each pair of homologous genes, the predicted protein sequences were used for multiple sequence alignment by MUSCLE (Edgar 2004) with default parameters, after which the nucleotide sequences were forced to fit the amino acid alignments by PAL2NAL (Suyama et al. 2006). *K*_s_ values were calculated using the Nei-Gojobori algorithm (Nei and Gojobori 1986) implemented in the codeml package of PAML (Yang 1997).

### Estimate of whole genome duplication timing

To time the Portulacineae WGD, we used the methods described in Supplemental material for the opium poppy genome (Guo et al. 2018). Briefly, we estimated the average evolutionary rate for Caryophyllales using common purslane, a Portulacineae and *A. hypochondriacus*, an Amaranthaceae. Given the mean *K_s_* value of common purslane-*A. hypochondriacus* and their divergence date T, we calculated the synonymous substitutions per site per year (r) for Caryophyllales (T = *K_s_*/2r). The r value and *K_s_* peak values of WGD were applied to time the common purslane WGD.

### Identification of C_4_-/CAM-specific PEPC genes

To identify PEPC genes in common purslane, we performed a BLASTP analysis using well-annotated PEPC genes in *A. thaliana* and maize as queries, as well as hidden Markov model searches using the profile PF00311 from the Pfam database as seed, against the genome-wide amino acid sequences of common purslane, employing BLASTP (e-value <1e−5) and the hmmsearch in HMMER (v3.1b2) (e-value <1e−03, –domE 0.001) (Potter et al. 2018), respectively. Then, multiple sequence alignment was conducted using MAFFT (v7.471) (Katoh et al. 2002), and gene trees were constructed using the maximum likelihood method in RAxML (v8.2.12) (Stamatakis 2014) with the “PROTGAMMAAUTO” model with 100 bootstrap replicates. Based on the previous classification studies in Caryophyllales (Christin et al. 2014), PEPC genes were divided into 7 categories.

### Gene coexpression and motif enrichment analysis

Transcription abundance from drought-treated and control samples were used to define condition-specific gene expression clusters. Genes with Transcripts Per Million (TPM) > 1 in at least 2 biological replicates from at least one condition were defined as expressed genes (30833 genes) and then log_2_-transformed TPM were used for coexpression analysis using the Weighted Gene Coexpression Network Analysis (WGCNA v1.70) (Langfelder and Horvath 2008) with signed network approach and a soft power threshold of 18. Minimal module size was set as 100 genes, initial module eigengenes with correlation coefficient >0.85 were merged, and 27 unique color-coded coexpression modules were produced. Cis-element enrichment analysis was performed as previously described (Rhie et al. 2020) with modifications. Briefly, genes with module membership >0.8 were selected, and 2000-bp upstream sequences from transcription start site were extracted. Occurrence of frequencies of the 656 known plant nonredundant motifs from JASPAR database (Castro-Mondragon et al. 2021) were determined by FIMO (v5.4.0) (Bailey et al. 2015) in each module as previously reported. The frequency of occurrence of motifs in promoters of all expressed genes was used as background. Enrichment analysis was performed using a Fisher's exact test with false discovery rate (FDR) correction (Yang et al. 2018), and motifs with FDR < 0.1 were defined as enriched individual motif. Enrichment score was calculated as log_2_ transformed ratio of occurrence frequency between coexpression module and genomic background. To reduce the redundancy of similar motifs, individual motifs were placed in 47 motif clusters determined by RSAT matrix-clustering (Castro-Mondragon et al. 2017), which were deposited on JASPAR website (https://jaspar.genereg.net/matrix-clusters/plants/).

## Accession numbers

The data generated in this study has been uploaded to the NCBI database and can be retrieved under accession numbers PRJNA978934 and PRJNA868526. The genome assembly and annotation have also been deposited in the Genome Warehouse in National Genomics Data Center under accession number GWHCBIU00000000 that is accessible at https://ngdc.cncb.ac.cn/gwh.

## Supplementary Material

kiad451_Supplementary_Data

## Supplemental data

The following materials are available in the online version of this article.

**Supplemental Figure S1.** Fluorescence in situ hybridization (FISH) of common purslane chromosomes with 45S rDNA and 5S rDNA.

**Supplemental Figure S2.** Estimation of common purslane genome size.

**Supplemental Figure S3.** Workflow of the de novo assembly of the common purslane genome.

**Supplemental Figure S4.** Hi-C contact matrix of the 26 pseudo-chromosomes for the common purslane assembly.

**Supplemental Figure S5.** LTR assembly index (LAI) assessment for each assembled common purslane chromosome.

**Supplemental Figure S6.** Estimation of the burst time of transposable elements in the common purslane genome.

**Supplemental Figure S7.** Syntenic blocks among the *B. vulgaris*, *H. undatus*, *P. amilis*, and *P. oleracea* genomes.

**Supplemental Figure S8.** Gene expression patterns of several key genes encoding enzymes or transporters identified in common purslane under drought and heat treatments during the day or night.

**Supplemental Figure S9.** Diagram depicting the gene copies encoding the main enzymes and transporters in the C_4_/CAM pathway and their expression levels under normal or stressful conditions.

**Supplemental Figure S10.** Expression patterns of C_4_-related copies and CAM-related gene copies in different tissues.

**Supplemental Figure S11.** Gene expression and differential expression patterns of PEPC genes identified in common purslane under heat and drought treatments during the day and night.

**Supplemental Figure S12.** RT-qPCR analysis of CAM-specific PEPC genes under control or stress treatments during the day (A) and at night (B).

**Supplemental Figure S13.** Heatmap of gene expression patterns of the 27 color-coded coexpression modules identified by WGCNA.

**Supplemental Figure S14.** Enrichment of cis-elements in day, night and drought-specific modules.

**Supplemental Figure S15.** Venn diagrams showing the numbers of shared and species-specific gene clusters in the 4 selected species.

**Supplemental Figure S16.** Analysis of whole-genome duplication and tandem duplication of the common purslane genome.

**Supplemental Figure S17.** Variation in gene copy number of several important gene families.

**Supplemental Figure S18.** Percentage of transcription factor genes within WGD and non-WGD genes.

**Supplemental Figure S19.** Percentage of transcription factor genes within TD and non-TD genes.

**Supplemental Figure S20.** An example of *HSF* genes resulting from WGD and TD in the common purslane genome that are upregulated upon stress treatment.

**Supplemental Table S1.** Summary statistics of contigs and scaffolds of the common purslane genome.

**Supplemental Table S2.** Mapping rates of RNA-seq and DNA-seq data to the assembled genome.

**Supplemental Table S3.** Benchmarking Universal Single Copy Orthologs (BUSCO) scores to assess the quality of the assembly and annotation.

**Supplemental Table S4.** Summary of the functional annotation for the annotated protein-coding genes.

**Supplemental Table S5.** Repeat contents in the common purslane genome.

**Supplemental Table S6.** Number of photosynthesis-related genes in different species.

**Supplemental Table S7.** Gene ID, species, and data source for all PEPC genes used in this study.

**Supplemental Table S8.** Summary of motif enrichment.

**Supplemental Table S9.** Assignment of genes into color-coded modules identified by WGCNA.

**Supplemental Table S10.** Genes with module membership > 0.8 in each module.

**Supplemental Table S11.** Significantly enriched motifs (FDR < 0.1) in each coexpression module.

**Supplemental Table S12.** Number of expanded and contracted gene families in the 16 species genomes analyzed in this study.

## Dive Curated Terms

The following phenotypic, genotypic, and functional terms are of significance to the work described in this paper:

TRB2 Gramene: AT5G67580
TRB2 Araport: AT5G67580

MYC2 Gramene: AT1G32640
MYC2 Araport: AT1G32640

PPDK Gramene: AT4G15530
PPDK Araport: AT4G15530
