# Supplementary material for: Gene duplications facilitate C4-CAM compatibility in common purslane
Source: Plant Physiol. 2023 Aug 17;193(4):2622–39. doi: 10.1093/plphys/kiad451 (PMC10663116; doi:10.1093/plphys/kiad451)
Supplement: kiad451_Supplementary_Data [file kiad451_supplementary_data.zip › Supplementary information.pdf]

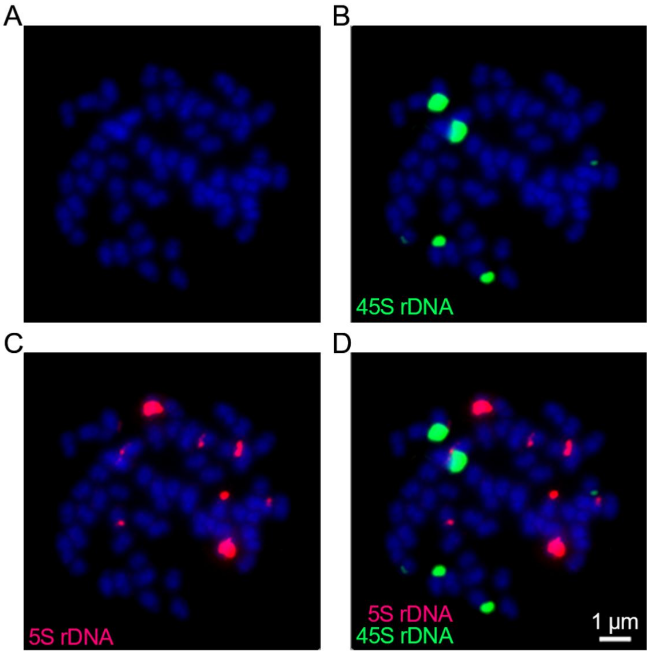

Supplemental Figure S1. Fluorescence *in situ* hybridization (FISH) of common purslane chromosomes with 45S rDNA and 5S rDNA.

A, *P. oleracea* chromosomes (blue) stained with 4′,6-diamidino-2-phenylindole (DAPI). **B,** FISH image of *P. oleracea* chromosomes hybridized with the 45S rDNA probe (green). **C,** FISH image of *P. oleracea* chromosomes hybridized with the 5S rDNA probe (red). **D,** Merged image from (B) and (C) demonstrating the overlapping signals from both probes. Scale bars, 1 μm.

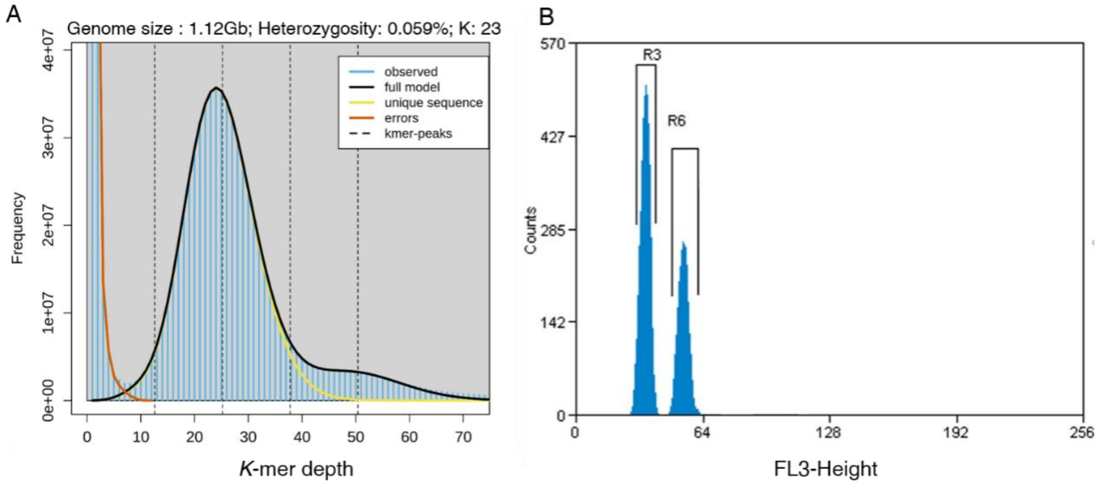

Supplemental Figure S2. Estimation of common purslane genome size.

A, *k*-mer analysis of genome size, with k = 23. Genome size (G) was estimated by G = k_{num}/k_{depth}, where k_{num} represents the total number of *k*-mers, and k_{depth} denotes the *k*-mer depth of the peak frequency of *k*-mer distribution. We also used GenomeScope to estimate the overall genome characteristics (heterozygosity rate, haploid genome size, and unique content) from Illumina data using Jellyfish v2.2.1014 with the parameters '-t 10 -C -m 19 -s 4G'. The total number of *k*-mers is 26,940,264,024, and the peak of the *k*-mer depth is 24; therefore, the estimated genome size is ~1,122 Mb. The genome of *P. oleracea* is mostly homozygous (with a heterozygosity rate of 0.0591%) and potentially in a diploid state (only one sharp peak was detected). **B,** Genome size estimation for *P. oleracea* (R6) based on flow cytometry using sorghum (R3, 732 Mb) as an internal reference. The genome size of *P. oleracea* was estimated to be ~1,137 Mb.

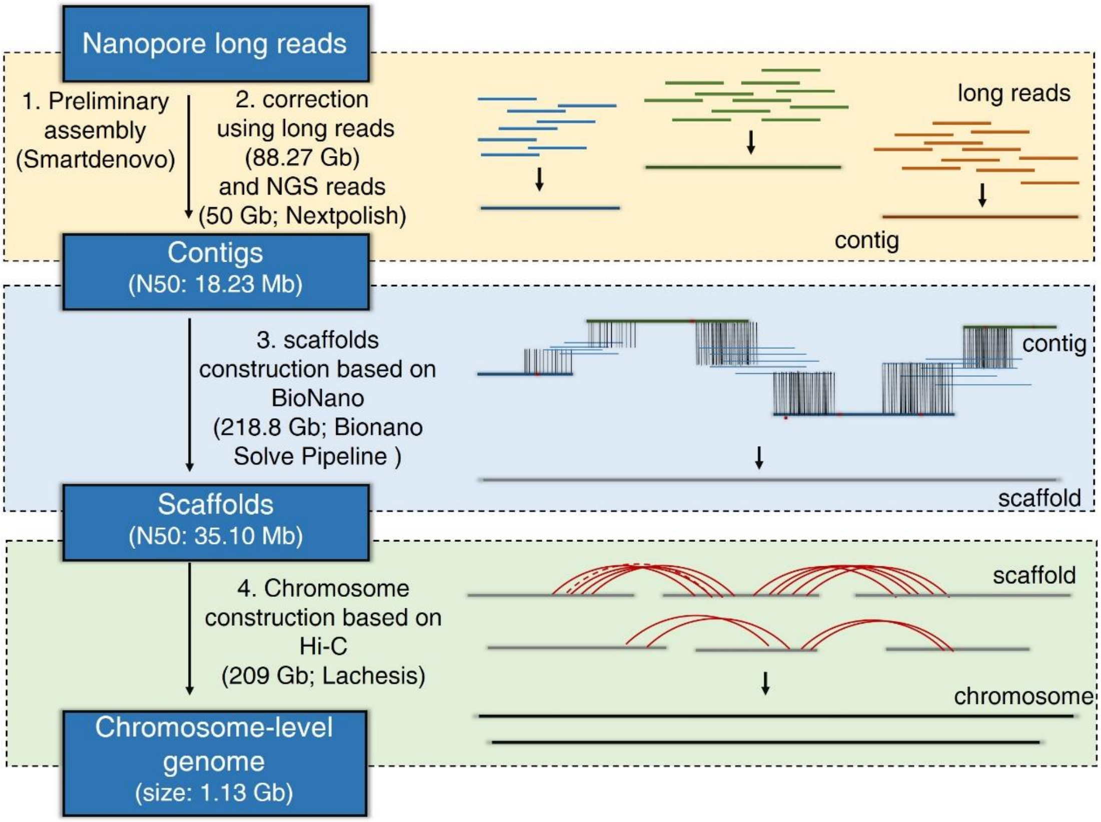

Supplemental Figure S3. Workflow of the *de novo* assembly of the common purslane genome.

Contigs were first obtained from overlapping long Nanopore reads. Then a scaffolding step was performed using Bionano optical maps. Finally, Hi-C data guided the chromosome-level genome assembly, based on interaction information within a chromosome.

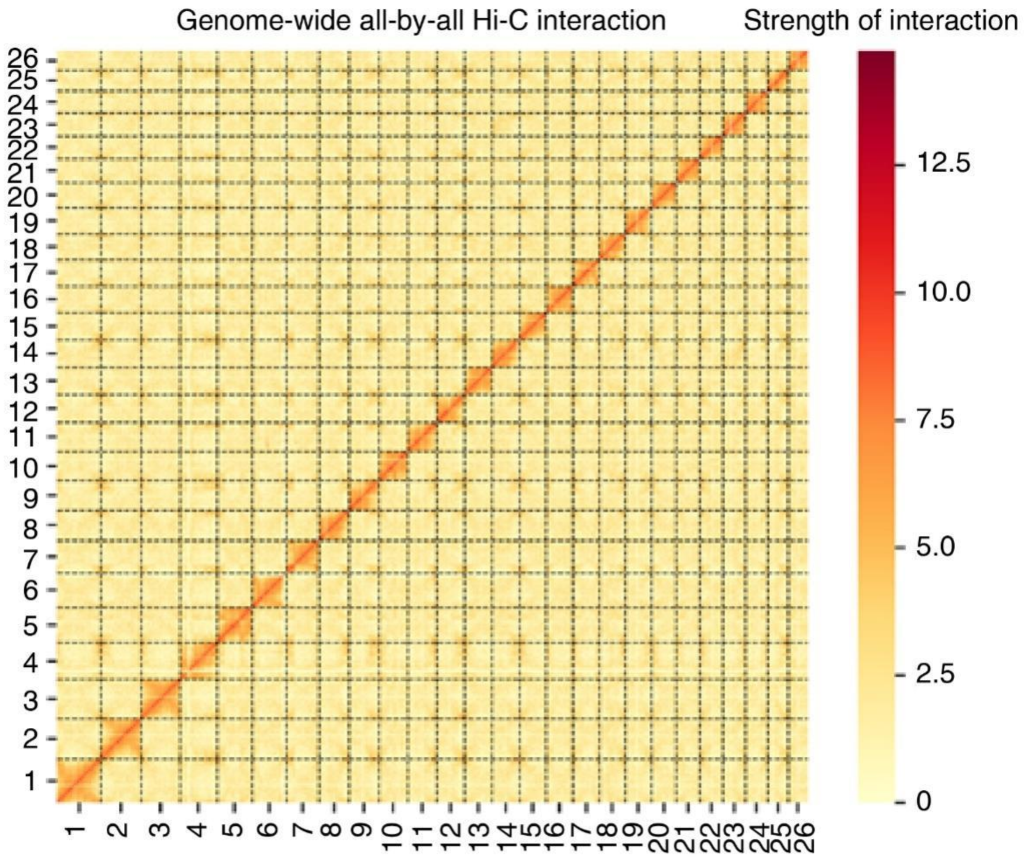

Supplemental Figure S4. Hi-C contact matrix of the 26 pseudo-chromosomes for the common purslane assembly.

The assembly results are consistent with the experimental results of FISH in terms of chromosome number.

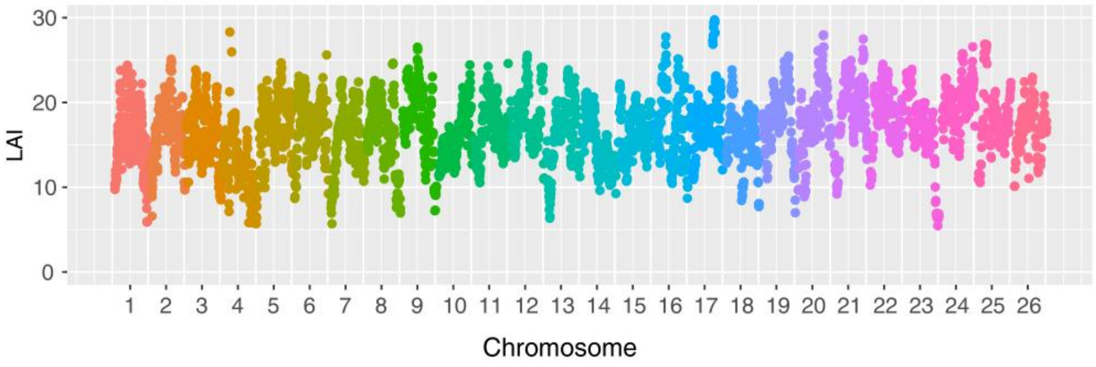

Supplemental Figure S5. LTR assembly index (LAI) assessment for each assembled common purslane chromosome.

The average LAI is about 17.96, reflecting the high quality of our assembly.

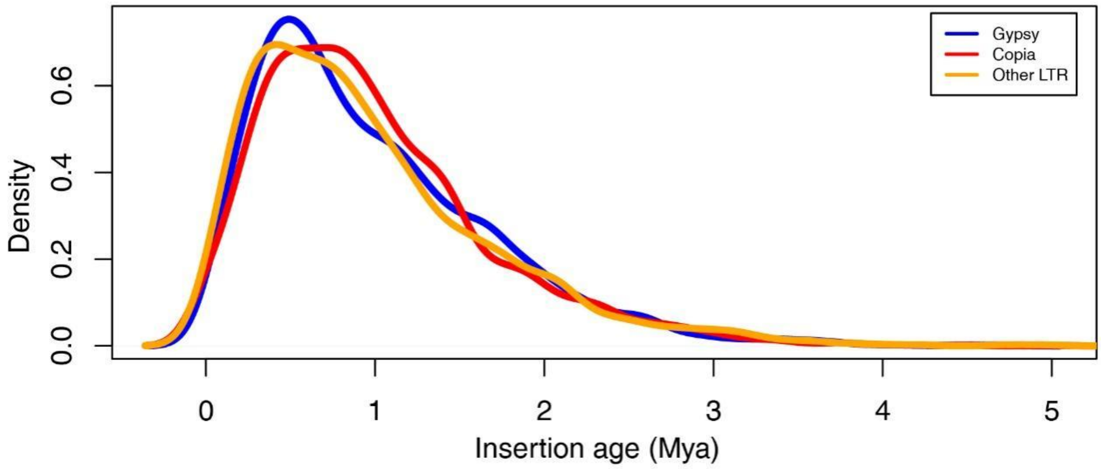

Supplemental Figure S6. Estimation of the burst time of transposable elements in the *P. oleracea* genome.

The distinct unimodal distribution for the insertion times of intact LTR-RTs in the *P. oleracea* genome suggests that the LTR burst occurred around 0.5 million years ago (Mya).

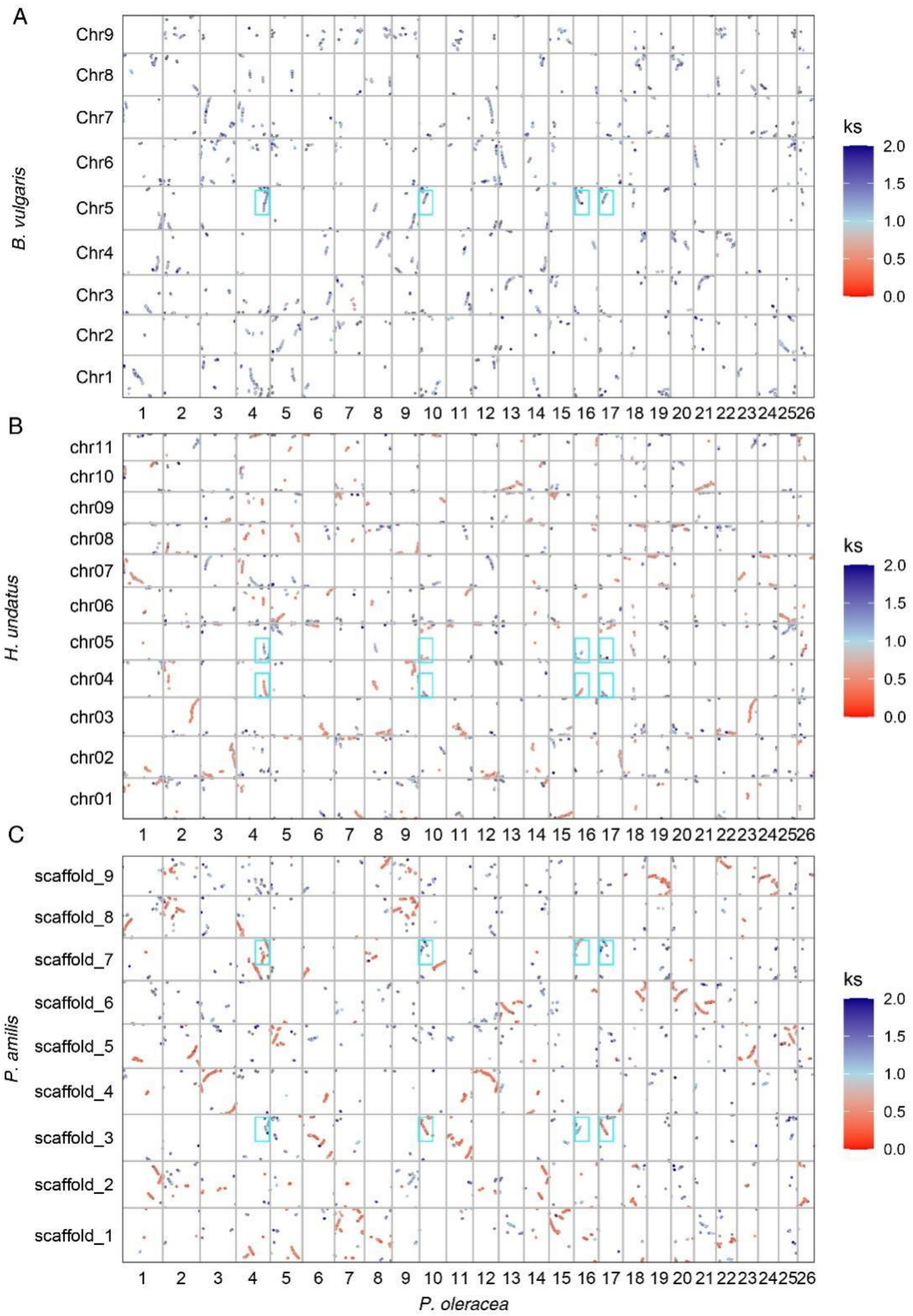

Supplemental Figure S7. Syntenic blocks among the *B. vulgaris*, *H. undatus*, *P. amilis*, and *P. oleracea* genomes.

A, Syntenic dotplot between the *P. oleracea* and *B. vulgaris* genomes. **B,** Syntenic dotplot between the *P. oleracea* and *H. undatus* genomes. **C,** Syntenic dotplot between the *P. oleracea* and *P. amilis* genomes. Dotplots show the orthologous regions of the relationship of 1:2:2:4.

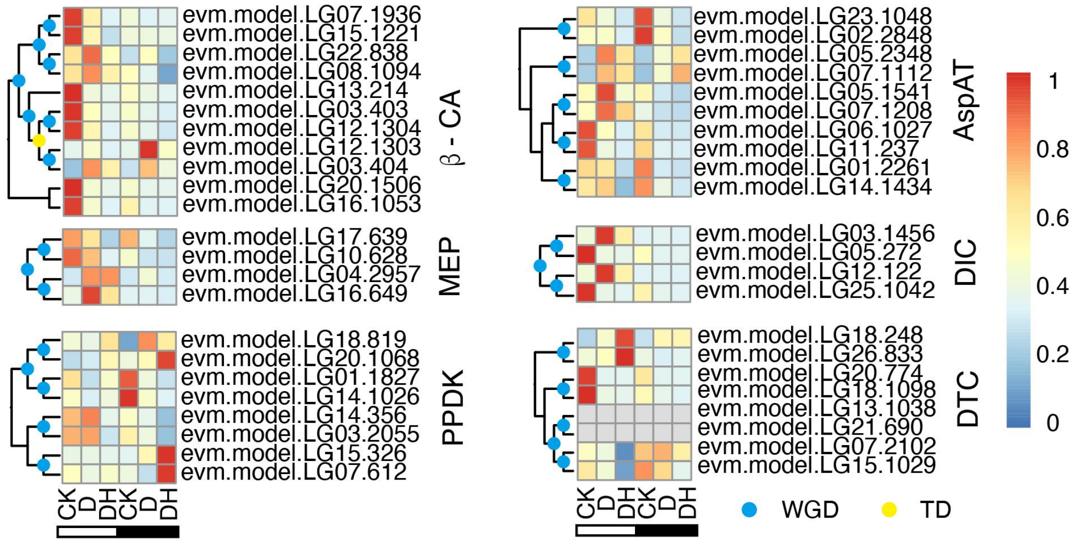

Supplemental Figure S8. Gene expression patterns of several key genes encoding enzymes or transporters identified in common purslane under drought and heat treatments during the day or night.

Cyan dots on the gene tree to the left indicate WGD events, while yellow dots represent TD events. CK, control group; D, drought group; DH, drought combined heat group. The expression level of each gene was normalized by the maximum value of TPM values.

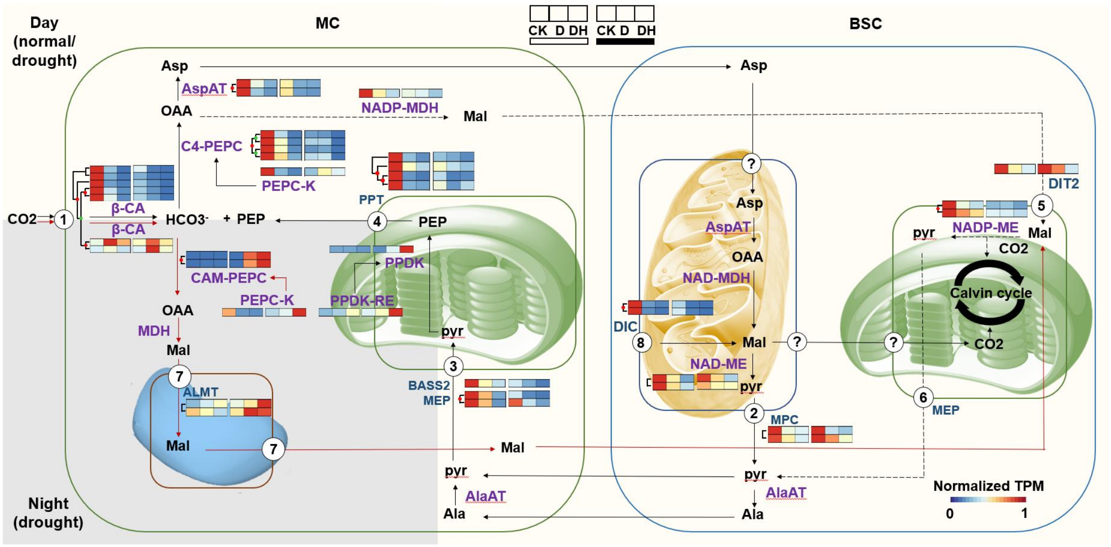

Supplemental Figure S9. Diagram depicting the gene copies encoding the main enzymes and transporters in the C_4/CAM pathway and their expression levels under normal or stressful conditions.

The light yellow background shows the normal C_4 pathway in *P. oleracea*; black arrows indicate metabolic fluxes (solid line, NAD-ME C_4 subtype; dashed line, potential NADP-ME subtype). The gray background and red arrows represent the nighttime CO_2 fixation pathway. Left box, mesophyll cell (MC); right box, bundle

sheath cell (BSC). Enzymes participating in photosynthesis are shown in purple, metabolites in black, and transporters in dark blue. Asp, aspartate; Mal, malate; pyr, pyruvate; OAA, oxaloacetate; PEP, phosphoenolpyruvate; β-CA, β-carbonic anhydrase; PEPC, phosphoenolpyruvate carboxylase; PPDK, pyruvate/orthophosphate dikinase; AspAT, aspartate aminotransferase; AlaAT, alanine aminotransferase; NAD(P)-MDH, NAD(P)-dependent malate dehydrogenase; NAD(P)-ME, NAD(P)-dependent malic enzyme; PEPC-K, PEPC kinase. Transporters are presented by circle number: 1, plasma membrane intrinsic protein (PIP); 2, mitochondrial pyruvate carrier (MPC); 3, sodium:pyruvate/proton:pyruvate cotransporter (BASS2/MEP); 4, PEP/phosphate translocator (PPT); 5, dicarboxylate transporter 2 (DIT2); 6. proton:pyruvate cotransporter (RER); 7, aluminum-activated malate transporter (ALMT); 8, dicarboxylate carrier (DIC). The numbers in brackets show C4- or CAM-related genes number/total number of gene family. CK, control group compared to the samples under certain stress; D, samples under drought; DH, samples under drought and heat. The expression level of each gene was normalized by the maximum value of TPM values.

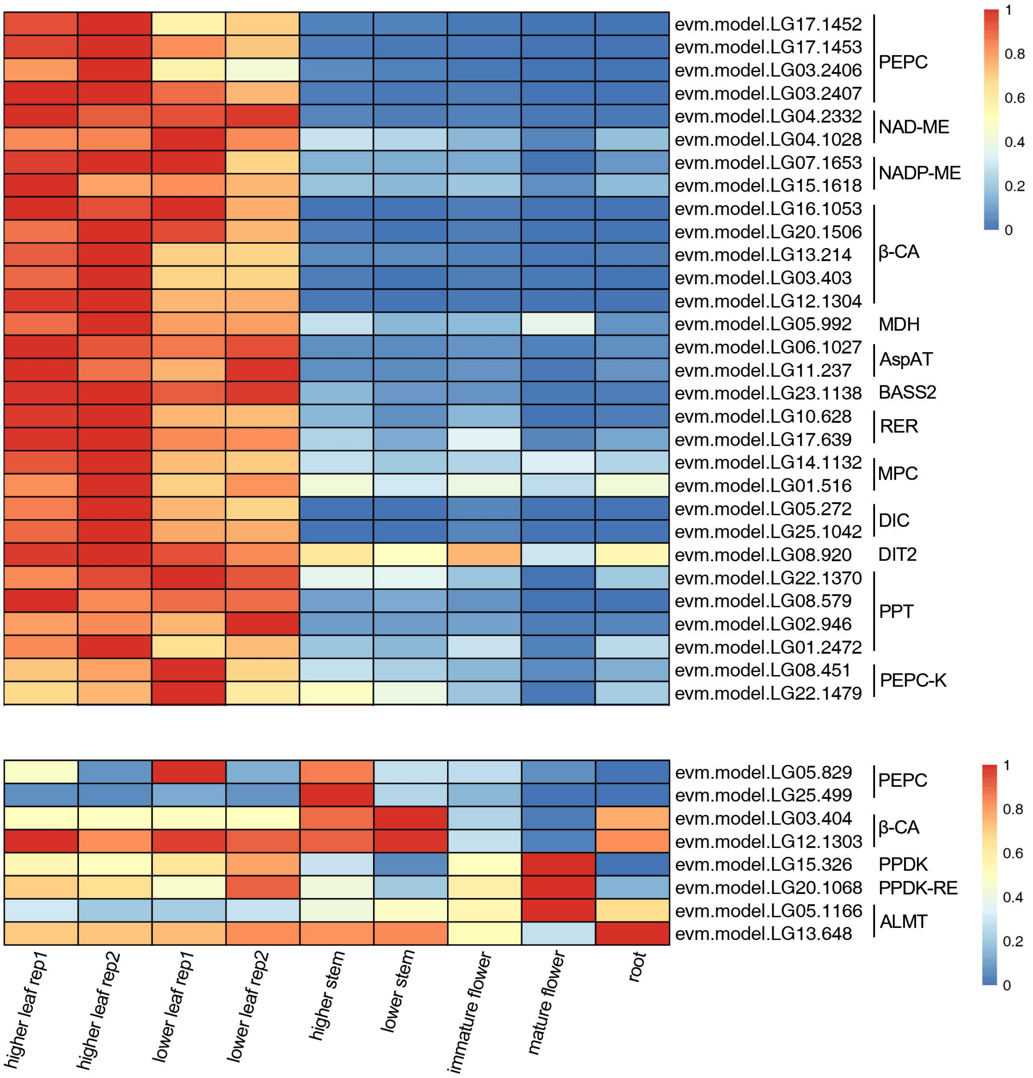

Supplemental Figure S10. Expression patterns of C_4-related copies and CAM-related gene copies in different tissues.

The expression level of each gene was normalized by the maximum value of all TPM values of the gene across all tissues.

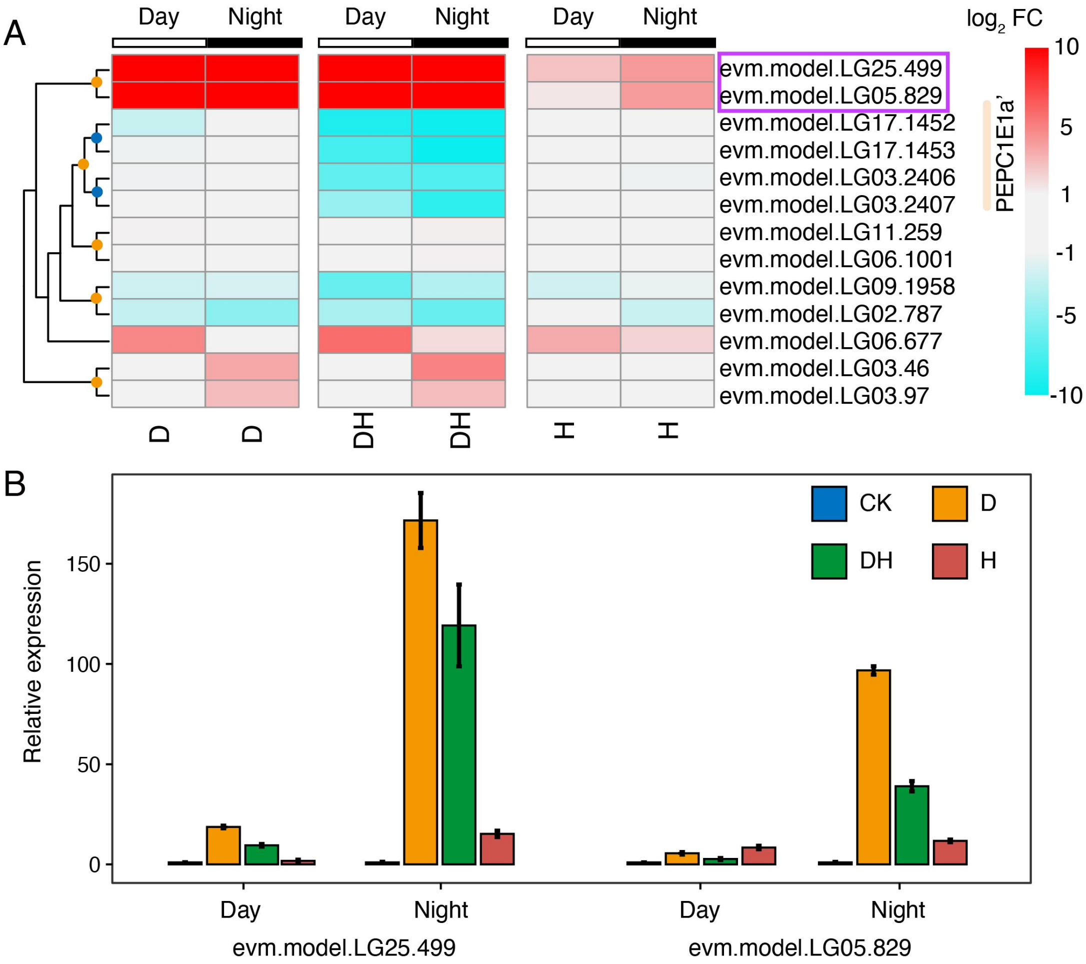

Supplemental Figure S11. Gene expression and differential expression patterns of PEPC genes identified in common purslane under heat and drought treatments during the day and night.

A, Differential expression patterns of PEPC genes**.** Orange dots in the gene trees to the left represent whole-genome duplication (WGD) events; blue dots represent tandem duplication (TD) events. CAM-specific PEPC genes are highlighted in magenta boxes. CK, control group; D, drought group; H, heat group; DH, drought combined heat group. B, RT-qPCR analysis of two PEPC1E1c genes exhibiting CAM-like expression patterns. Error bars represent standard error.

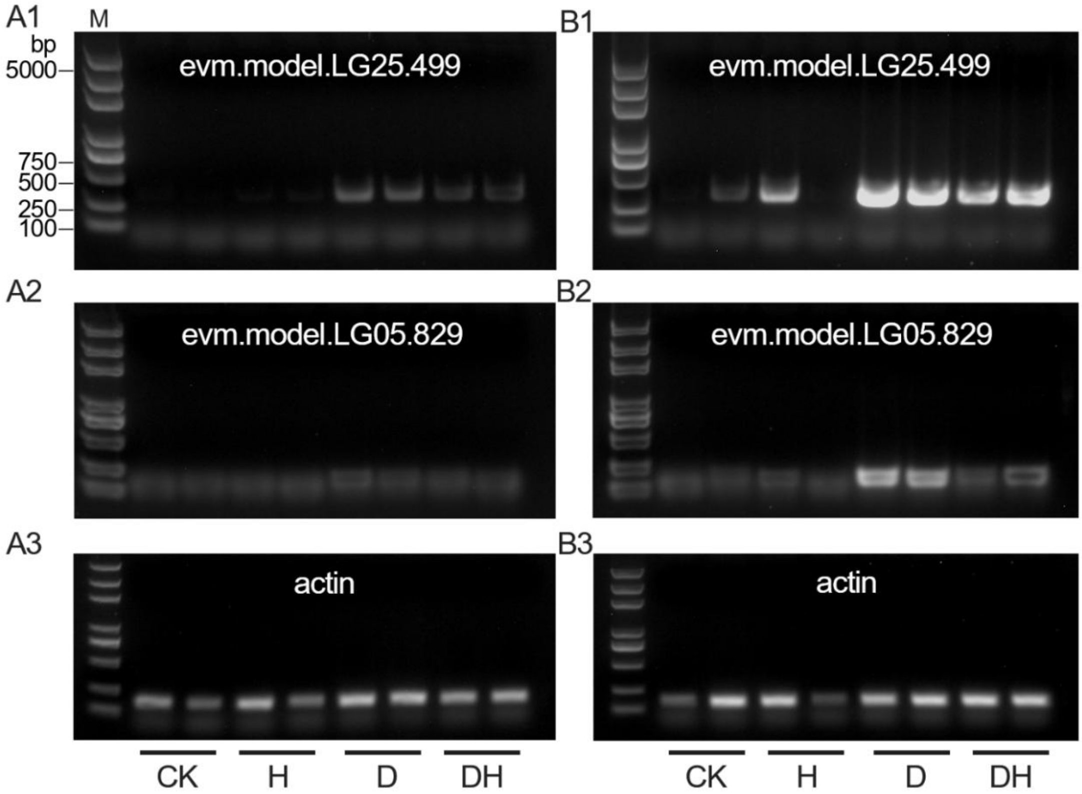

Supplemental Figure S12. RT-qPCR analysis of CAM-specific *PEPC* genes under control or stress treatments during the day (A) and at night (B).

CK, control group; D, drought group; H, heat group; DH, drought combined heat group.

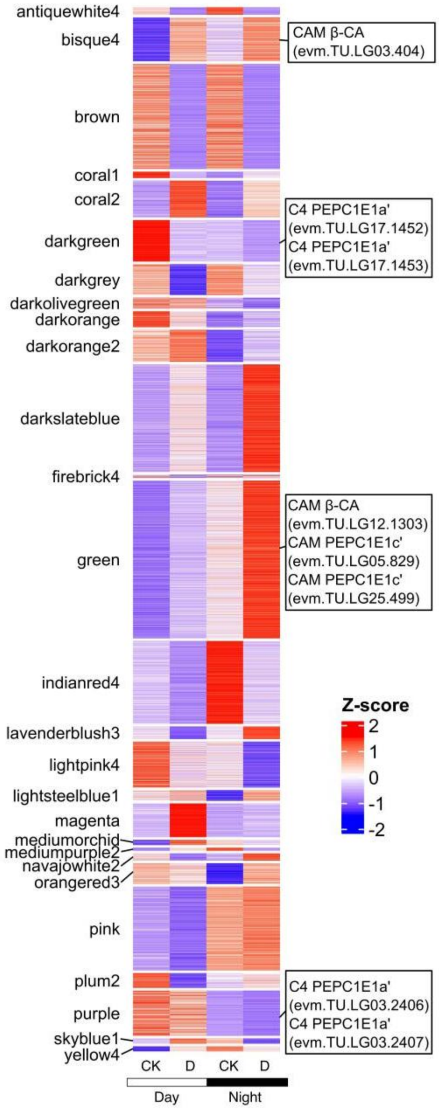

Supplemental Figure S13. Heatmap of gene expression patterns of the 27 color-coded co-expression modules identified by WGCNA.

Data are presented as Z-score. C_4 and CAM specific genes were indicated.

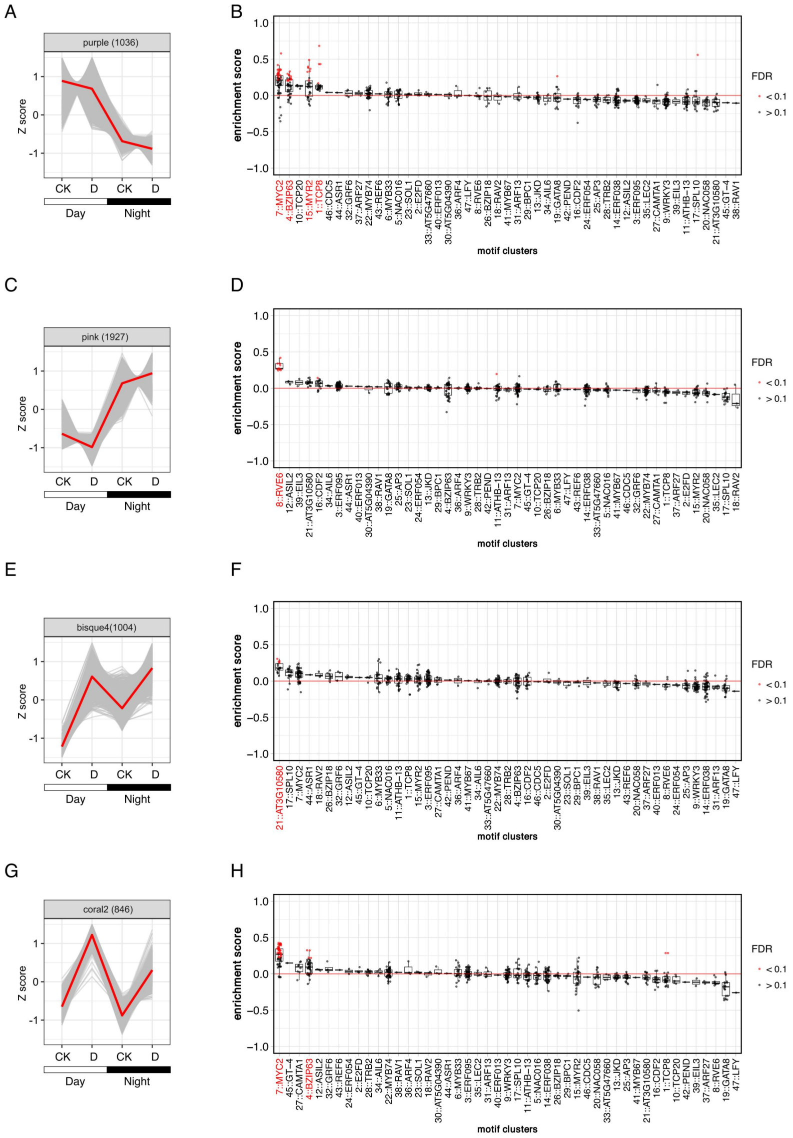

Supplemental Figure S14. Enrichment of cis-elements in day, night and drought specific modules.

(A, C, E, G) line plots showing expression pattern of day (A), night (C) and drought (E, G) specific modules, data are presented as Z-score, mean of expression is presented as red lines. (B, D, F, H) enriched motifs in promoters from genes in day (B), night (D) and drought (F, H) specific modules presented in motif clusters, significant motifs were indicated in red dots, motif clusters with more than three enriched motifs were highlighted in red text.

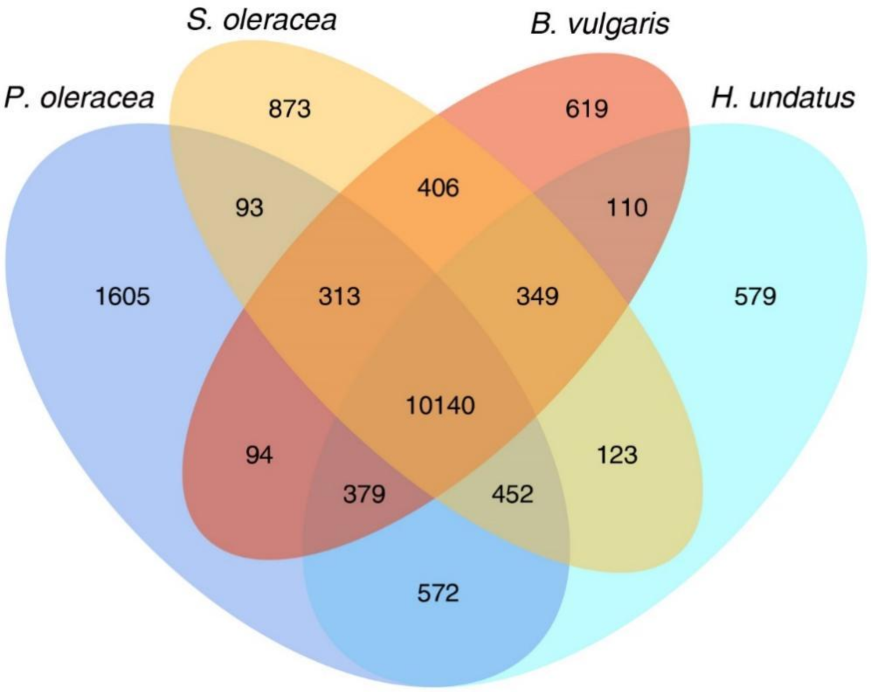

Supplemental Figure S15. Venn diagrams showing the numbers of shared and species-specific gene clusters in the four selected species.

The *P. oleracea* genome possesses the largest number of species-specific gene clusters compared to its relative species.

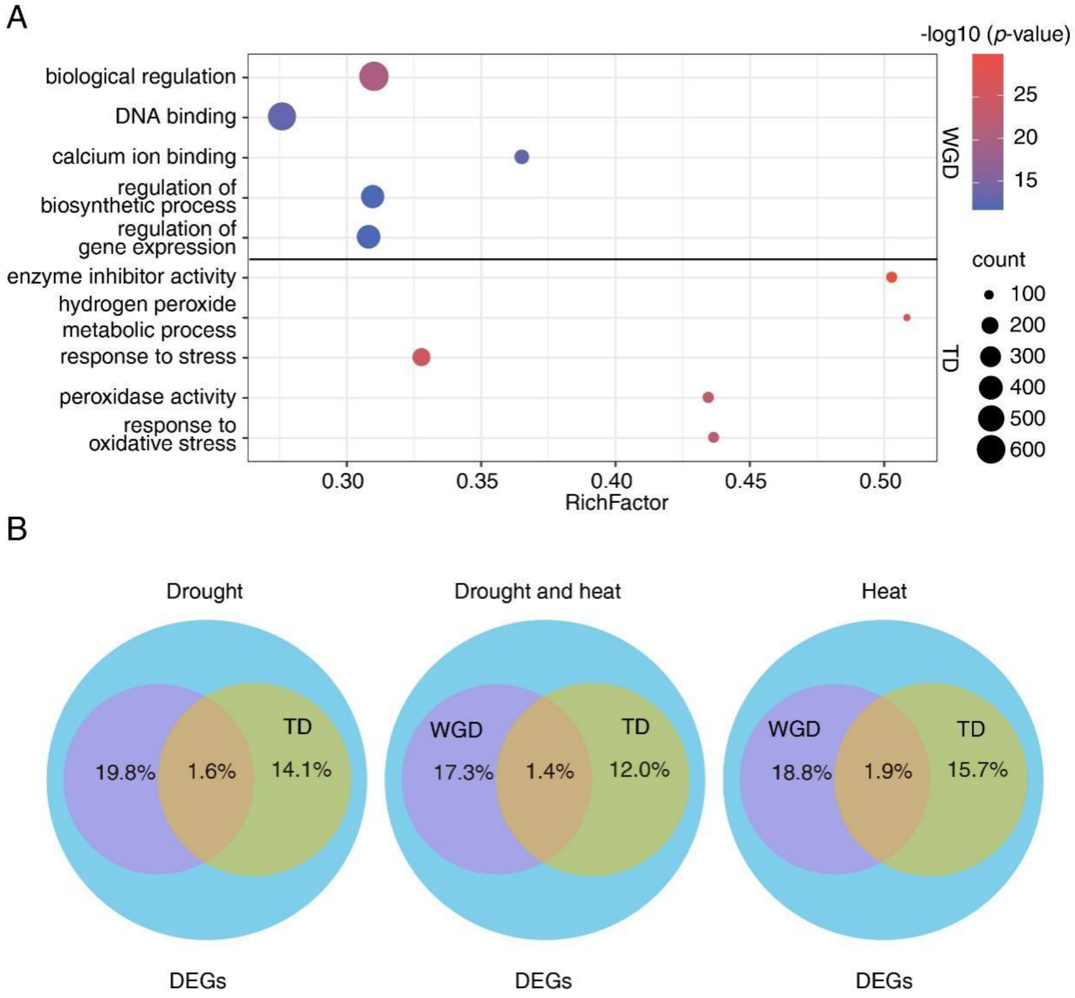

Supplemental Figure S16. Analysis of whole-genome duplication and tandem duplication of the common purslane genome.

A, Significantly enriched GO terms in genes having undergone WGD and TD in the *P. oleracea* genome. The results are sorted according to significance. **B,** PropVenn diagrams showing the extent of overlap between differentially expressed genes (DEGs) having arisen from WGD, TD, both, or neither.

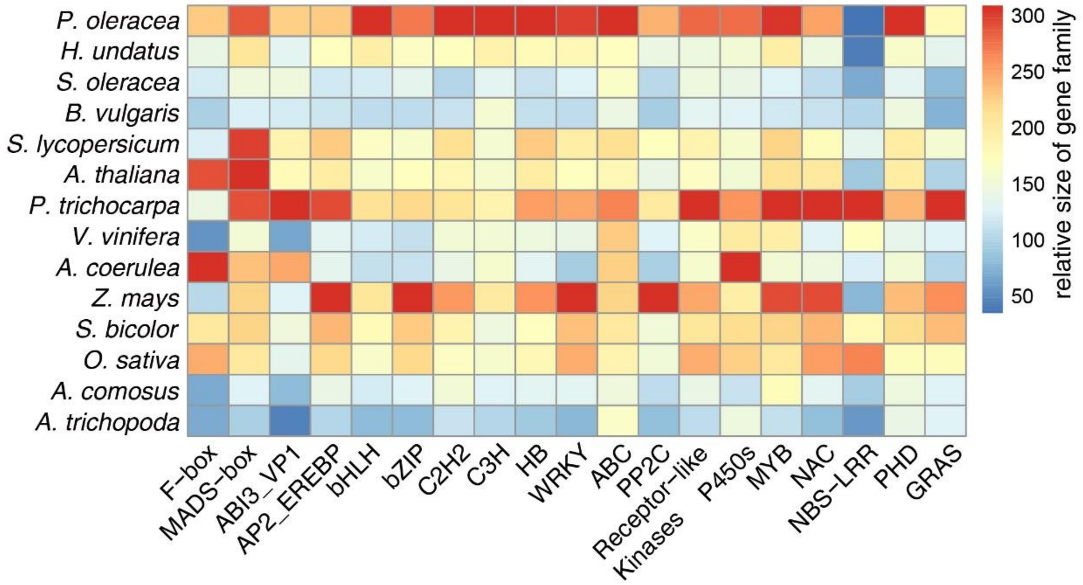

Supplemental Figure S17. Variation in gene copy number of several important gene families.

These gene families have been described previously by the OneKP Project (One Thousand Plant Transcriptomes Initiative, 2019)

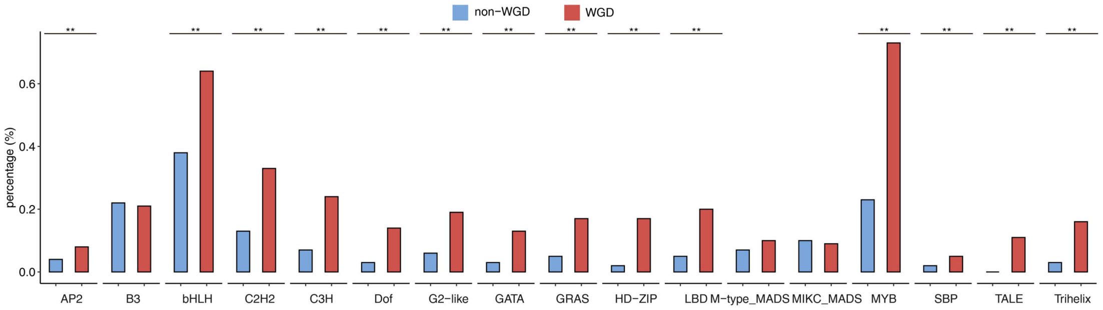

Supplemental Figure S18. Percentage of transcription factor genes within WGD and non-WGD genes.

** $P < 0.01$ by chi-square test.

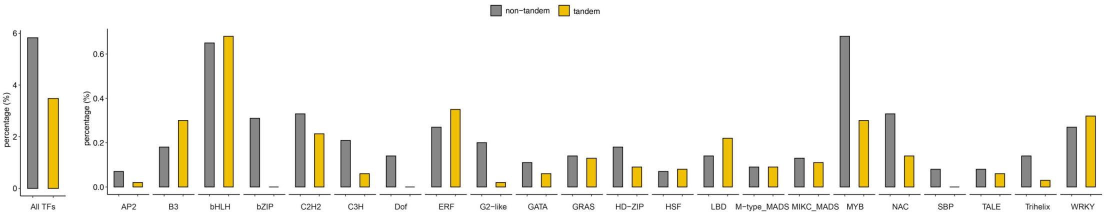

Supplemental Figure S19. Percentage of transcription factor genes within TD and non-TD genes.

None of these TF families were significantly enriched in the TD gene sets, as determined by a chi-square test.

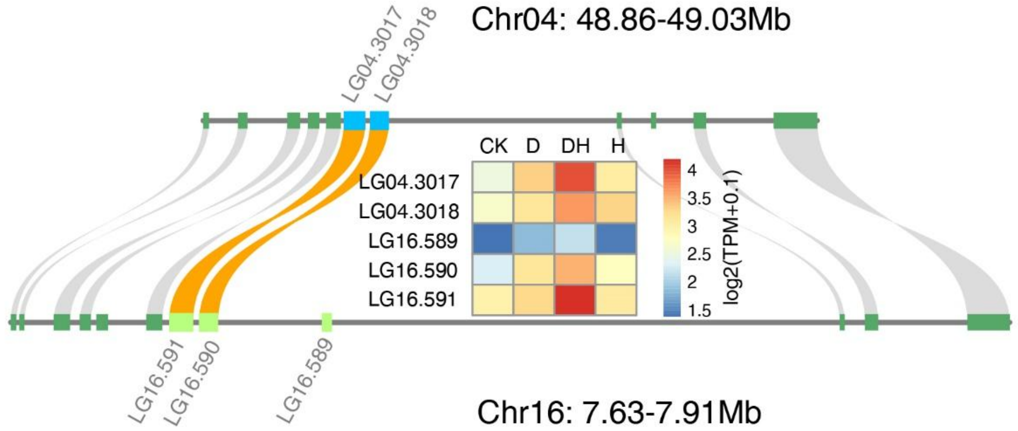

Supplemental Figure S20. An example of *HSF* genes resulting from WGD and TD in the common purslane genome that are upregulated upon stress treatment. WGD genes are connected by curved lines, and TD genes are marked in the same color but not connected on the same chromosome. CK, control group; D, drought group; H, heat group; DH, drought combined heat group.
